# Left ventricular wall thickness heterogeneity improves cardiovascular disease diagnosis and prognosis: a UK Biobank cardiovascular magnetic resonance cohort study

**DOI:** 10.1093/ehjimp/qyaf092

**Published:** 2025-07-18

**Authors:** Kerrick Hesse, Mohammed Y Khanji, C Anwar A Chahal, Steffen E Petersen, Nay Aung

**Affiliations:** William Harvey Research Institute, NIHR Barts Biomedical Research Centre, Queen Mary University London, Charterhouse Square, London EC1M 6BQ, UK; Academic Cardiovascular Unit, James Cook University Hospital, Marton Road, Middlesbrough TS4 3BW, UK; William Harvey Research Institute, NIHR Barts Biomedical Research Centre, Queen Mary University London, Charterhouse Square, London EC1M 6BQ, UK; Newham University Hospital, Barts Health NHS Trust, Glen Road, Plaistow, London E13 8SL, UK; Barts Heart Centre, St Bartholomew’s Hospital, Barts Health NHS Trust, West Smithfield, London EC1A 7BE, UK; Barts Heart Centre, St Bartholomew’s Hospital, Barts Health NHS Trust, West Smithfield, London EC1A 7BE, UK; Center for Inherited Cardiovascular Diseases, WellSpan Health, Lancaster, PA, USA; Cardiac Electrophysiology, Cardiovascular Division, Hospital of the University of Pennsylvania, Philadelphia, PA, USA; Department of Cardiovascular Medicine, Mayo Clinic, Rochester, MN, USA; William Harvey Research Institute, NIHR Barts Biomedical Research Centre, Queen Mary University London, Charterhouse Square, London EC1M 6BQ, UK; Barts Heart Centre, St Bartholomew’s Hospital, Barts Health NHS Trust, West Smithfield, London EC1A 7BE, UK; William Harvey Research Institute, NIHR Barts Biomedical Research Centre, Queen Mary University London, Charterhouse Square, London EC1M 6BQ, UK; Barts Heart Centre, St Bartholomew’s Hospital, Barts Health NHS Trust, West Smithfield, London EC1A 7BE, UK

**Keywords:** biomarker, cardiovascular magnetic resonance imaging, heterogeneity, left ventricular hypertrophy, wall thickness

## Abstract

**Aims:**

Left ventricular hypertrophy (LVH) regionality carries diagnostic and prognostic importance. Mean absolute deviation of maximum segmental wall thickness (*MadWT*) is a novel left ventricular wall thickness (LVWT) heterogeneity biomarker from cardiovascular magnetic resonance imaging (CMR).

**Objectives:**

To compare *MadWT* to indexed LV mass (*LVMi*), maximum (*MaxWT*) and mean (*MeanWT*) wall thickness to predict incident cardiovascular disease (CVD) and differentiate physiological from pathological LVH in highly physically active individuals.

**Methods and results:**

Deep learning-assisted analysis of 44 930 UK Biobank CMR scans produced WT indices. Cox regression modelled major adverse cardiovascular events (MACE), heart failure (HF), arrhythmia, and all-cause death against LVWT indices. In the top 1% most physically active biomarker differences between propensity score matched hypertensive and non-hypertensive groups were compared. Over median (Q1, Q3) follow-up of 5.7 (4.9, 7.1) years, *MadWT, MaxWT, MeanWT*, and *LVMi* were associated with greater risk of MACE, HF, arrhythmia (*P* < 0.05), but not all-cause death (*P* > 0.05). After adjusting for CMR biomarkers, including *LVMi*, *MadWT* remained independently prognostic of the greatest number of endpoints, including MACE, HF, and arrhythmia [HR 1.13 (1.04–1.23); HR 1.15 (1.01–1.32); and HR 1.26 (1.18–1.35) respectively]. In the top 1% most physically active by three metrics, *MadWT* was the only significantly different biomarker between hypertensive and non-hypertensive participants (*P* < 0.05).

**Conclusion:**

*MadWT* is important prognostically beyond LV mass and may be useful when differentiating physiological from hypertensive LVH. Although findings require confirmation in athletic and diseased cohorts, *MadWT* is readily translatable to deep learning-assisted clinical CMR reporting, especially in early unexplained LVH.

## Graphical Abstract

**Graphical Abstract:**
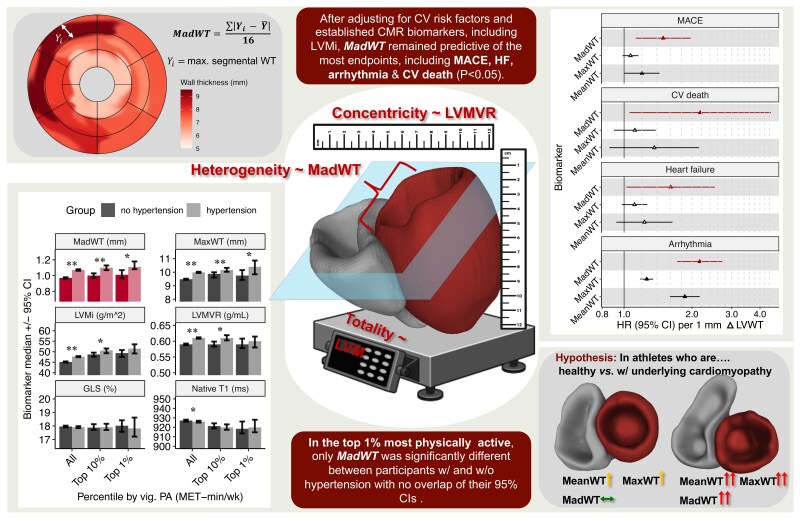
*MadWT* assesses LV wall thickness heterogeneity. *MadWT* quantifies LV wall thickness heterogeneity, a characteristic of LVH not captured by conventional indices, including *LVMi* and *LVMVR*. The marker (i) prognosticates independently and incrementally ischaemic and HF risk above conventional CV risk factors and CMR biomarkers; and (ii) facilitates the differentiation between physiological and pathological LVH in intermediate phenotypes. CI, confidence interval; CMR, cardiovascular magnetic resonance imaging; CV, cardiovascular; GLS, global longitudinal strain; HF, heart failure; HR, hazard ratio; LVH, left ventricular hypertrophy; LVMi, indexed left ventricular mass; LVMVR, left ventricular mass to volume ratio; MACE, major adverse cardiovascular events; MadWT, mean absolute deviation of maximum segmental wall thickness; MaxWT, maximum end-diastolic wall thickness; MeanWT, mean wall thickness; MET, metabolic equivalent of task; PA, physical activity; SD, standard deviation; WT, wall thickness.

## Introduction

Left ventricular hypertrophy (LVH) broadly describes elevated LV mass (LVM). Increased LVM, as measured by electrocardiography (ECG), echocardiography and cardiovascular magnetic resonance imaging (CMR), is independently associated with a greater risk of major adverse cardiovascular events (MACE).^1–3^

More detailed characterisation of LVH by the presence of LV dilatation (concentric vs. eccentric), LV wall thickness (LVWT) symmetry and involvement of the right ventricle (RV) stratifies prognosis further.^1,4,5^ In a Multi-Ethnic Study of Atherosclerosis (MESA) CMR study, elevated LV mass to volume ratio (LVMVR), a measure of concentric remodelling, was more predictive of future atherothrombotic events, including myocardial infarction (MI) and stroke than LVM.^1^ Currently, there is no imaging biomarker that comprehensively measures global heterogeneity of LVWT; increased heterogeneity may reflect patchy localized remodelling and fibrosis that is either not detectable or missed by conventional CMR indices, particularly on unenhanced cine sequences.

Furthermore, LVH in the athletic individual remains a diagnostic challenge.^6,7^ The myocardium adaptively thickens in response to increased physiological (e.g. weightlifting), pathological (e.g. hypertension) pressure overload and, less commonly, intrinsic myocardial disease [e.g. hypertrophic cardiomyopathy (HCM)].^8,9^ CMR can reliably differentiate aetiologies of severe LVH by unique tissue signatures on different sequences, including late gadolinium enhancement (LGE) and native T1 mapping-derived extracellular volume.^4,10–13^ However, these patterns may not be seen in milder LVH phenotypes, for example, in HCM gene-positive athletes with LVWT <15 mm.^9^ Instead, LVWT heterogeneity may be important by differentiating the homogeneous remodelling of physiological LVH from the often asymmetrical wall thickening associated with pathological LVH.^14–16^

**Figure 1:**
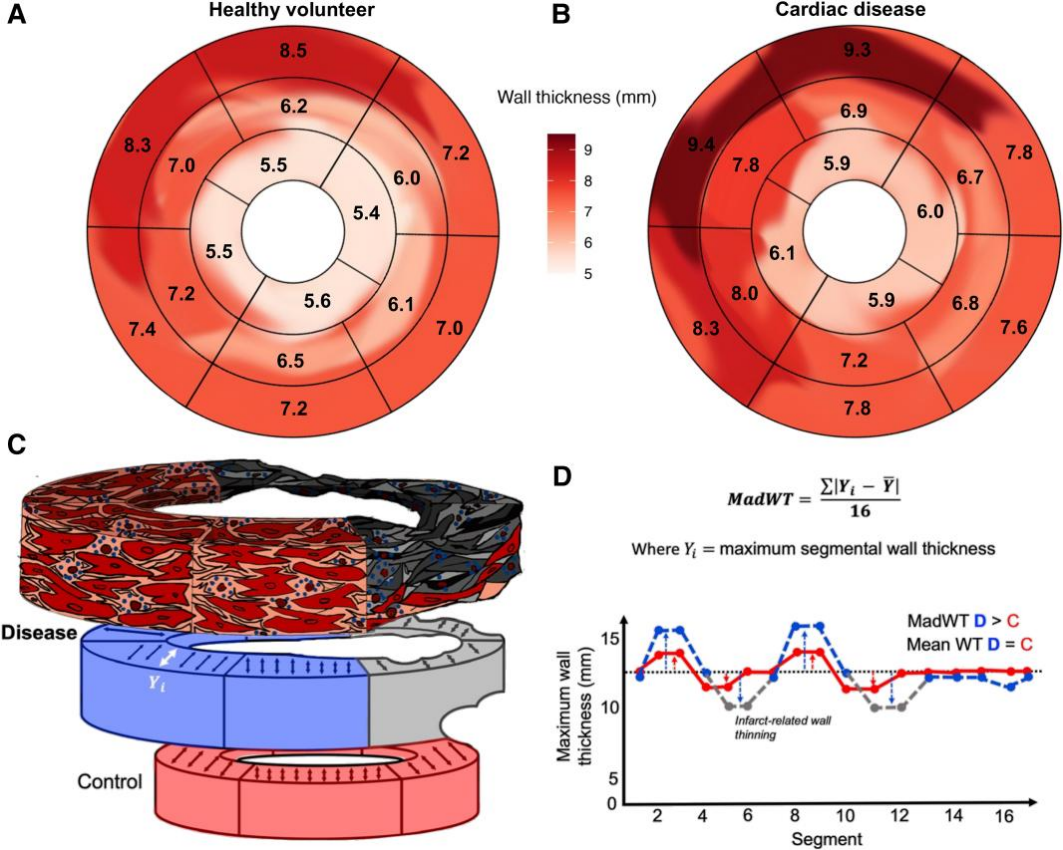
Segmental wall thickness heterogeneity and *MadWT*. Heterogeneity in maximum segmental wall thickness is visibly greater in UK Biobank participants with (Panel *B*) vs. without (Panel *A*) established cardiac disease. On the historical American Heart Association 16-segment left ventricular model, mean absolute deviation of *MadWT* quantifies WT heterogeneity by first, calculating differences between maximum segmental and mean WT and second, deriving the mean of the 16 differences (Panels *C* and *D*).

Only LVM and maximum WT are routinely measured on CMR despite the significance of LVH heterogeneity and the proliferation of reliable deep learning-derived segmentation algorithms. Preliminary automated CMR image analysis of ∼45 000 UK Biobank participants demonstrated noticeably greater maximum segmental WT variation in participants with vs. without cardiac disease (*Figure 1A* and *B*). We propose mean absolute deviation of maximum segmental wall thickness (*MadWT*) at end-diastole as a novel WT heterogeneity biomarker. On a 16-segment LV model, WT heterogeneity is quantified by calculating differences between maximum segmental and mean WT and deriving the mean of these 16 delta values (*Figure 1C* and *D*).

Our goal is to explore *MadWT* by investigating its predictors, prognostic importance, and relationship to physical activity and hypertension-related cardiac remodelling. In an above-average physically active cohort from the UK Biobank, we aimed to compare *MadWT* to maximum end-diastolic WT (*MaxWT*), mean end-diastolic WT (*MeanWT*), and indexed LV mass (*LVMi*), to discriminate between physiological and pathological LVH and predict MACE.

## Methods

### Study population

Data was sourced from the UK Biobank (UKBB) under application number 2964. The UKBB resource is a prospective cohort study of 500 000 people aged 40–69 years at enrolment, recruited between 2006 and 2010 across the UK with detailed phenotype and genotype characteristics and indirectly followed-up through active outcome accrual from disease registries, primary care records and hospital episodes.^17^ All participants provided written consent to participate and publish anonymized health-related data during recruitment. The North West Multi-centre Research Ethics Committee provided ethical approval, most recently under 21/NW0157.^18^ The study was limited to participants with available CMR from the UKBB imaging sub-study.^19,20^

### CMR protocol

As part of a multi-modality enhancement visit, one-fifth of UKBB participants underwent CMR. The protocol rationale and design have been described previously.^20,21^ Relevant sequences from the 20-min protocol included (i) bright-blood anatomic assessment in the sagittal, coronal, and axial planes; (ii) balanced steady-state free precession (bSSFP) cines of the ventricles and atria including three long axis (LAX) and a complete short axis (SAX) stack and (iii) one SAX native T1 mapping slice mid-ventricle on a 1.5 T scanner (MAGNETOM Aera, Syngo Platform VD13A, Siemens Healthcare, Erlangen, Germany).^21^

### CMR analysis and quality control

The quality control process for CMR acquisition and analysis has been described elsewhere.^20,21^ CVI42 post-processing software (Version 5.14.1, Circle Cardiovascular Imaging Inc., Calgary, Canada) facilitated large-volume batch analysis of CMR scans. We used a modified automatic image analysis pipeline based on a fully convolutional network trained and tested against 4875 expert-annotated CMR scans to measure standard volumetric and functional parameters on bSSFP cines.^22,23^ In brief, the algorithm automatically contoured the LV epi- and endocardium, segmented the myocardium into 16 segments and measured the maximum distance between the two layers per segment in the end-diastolic phase on the SAX bSSFP cine sequences.^23^ Quantitative evaluation of the automated method included mean Dice values and mean contour distance 0.94, 1.04 mm for LV cavity and 0.88, 1.14 mm for LV myocardium, demonstrating good agreement between manual and automatic segmentation.^22^ Additionally, we applied a feature tracking-based machine learning algorithm to said sequences to quantify longitudinal and circumferential strain.^24^ Average regional native T1 was derived from a mid-ventricular SAX slice with 10% epi- and endocardial boundary offsets to minimize the risk of partial volume effects.

The minimum dataset from each CMR scan included the dimension indices *MadWT* (mm), *MaxWT* (mm) and *MeanWT* (mm) as well as the volumetric parameters indexed LV end-diastolic volume [*LVEDVi* (mL/m^2^)], *LVMi* (g/m^2^), LV mass to volume ratio [*LVMVR* (g/mL)] and LV ejection fraction [*LVEF* (%)]. Based on a 16-segment LV model, *MeanWT* was the average maximum segmental WT, and *MaxWT* was the maximum segmental WT at end-diastole. Segmental WT measurements that were ≥3 × interquartile below 25th percentile i.e. above 75th percentile were removed in line with a statistical outlier quality control approach validated against trained physician CMR contouring and segmentation.^24^ Participants with <12 segmental WT measurements were excluded to reduce measurement bias (see Supplementary data online, *Figure S1*).

### Phenotype definitions

We defined clinical phenotypes using a combination of medical codes derived from the International Classification of Diseases (ICD-9 and -10) and the Operating Procedure Codes Supplement (OPCS-3 and -4), self-reported diagnoses, disease-specific medications, and biochemical profiles. Supplementary data online, *Table S1* lists the definitions of relevant cardiometabolic risk factors and cardiovascular diseases (CVD). Individuals with prevalent CVD were excluded from regression analyses. Remaining participants were dichotomized into healthy reference and CV risk factor (CVRF) cohorts based on smoking status and the presence of hypertension, hyperlipidaemia, diabetes, and obesity.

Primary endpoints were incident HF, arrhythmia, including atrial fibrillation (AF), ventricular arrhythmia, and bradyarrhythmia, death from any cause, and MACE. Secondary endpoints were the components of MACE, including MI, stroke, and CV death.

### Level of physical activity

Data from physical activity (PA) questionnaires and wrist-worn accelerometers were leveraged to quantify participant PA levels. The UKBB accelerometer sub-study has been described previously; we used the mean acceleration vector (milli-gravity) from participants with at least 72 h of calibrated wear data.^25^ From PA questionnaires, we derived total PA in metabolic equivalent of task units (MET) per week as below:^26,27^


$$\begin{array}{rl} \mathrm{TPA}\left(\mathrm{MET}-\frac{\min}{\mathrm{week}}\right) & =3.3\;\times \mathrm{walking}\;\mathrm{minutes}\;\times \mathrm{days}\;\\ & \;\;\;+4.0\;\times \mathrm{moderate}\;\mathrm{intensity}\;\mathrm{minutes}\;\times \mathrm{days}\;\\ & \;\;\;+8.0\;\times \mathrm{vigorous}\;\mathrm{intensity}\;\mathrm{minutes}\;\times \mathrm{days} \end{array}$$

### Statistical analysis

We summarized numerical variables with mean ± standard deviation (SD) or median [interquartile range (IQR)] depending on the normality of data distribution (see Supplementary data online, *Figure S2*). Categorical variables were reported as proportions. Inter-group differences were assessed with the Student’s *t*-test i.e. Mann–Whitney *U* test and Fisher’s exact test.

To investigate the association between *MadWT* and CVRF, we fitted a generalized linear model (GLM) with an ‘*identity’* link function and a *Gamma* distribution. The outcome variable was *MadWT*; predictor variables included age, sex, ethnicity, height, body mass index (BMI), PA level, smoking, hyperlipidaemia, hypertension, and diabetes status.

Cox proportional hazards regression methodology modelled incidence of previously defined clinical outcomes against *MadWT, MaxWT*, *MeanWT*, and *LVMi*. We constructed models in a stepwise fashion, starting with (Model 1) univariable analysis before sequentially adding (Model 2) CVRF (i.e. age, sex, ethnicity, BMI, smoking status, hyperlipidaemia, hypertension and diabetes) and (Model 3) standard CMR biomarkers (i.e. *LVEDVi* and *LVEF*). Hazard ratios (HR) with 95% confidence intervals (CI) estimated biomarker effect sizes. Sensitivity analyses were performed in men and women separately. In an exploratory regression Model 4, outcomes were regressed against WT indices after additionally adjusting for *LVMi.* Biomarker effect sizes were reported per unit and per SD as described. Variance inflation factors (VIF) (see Supplementary data online, *Table S2*) and *Schoenfield* residual vs. time plots (see Supplementary data online, *Figures S3*–*S9*) demonstrated absence of multicollinearity (VIF < 5.0) and agreement with the proportional hazards assumption respectively.

Finally, in propensity score matched (PSM) cohorts of participants with and without hypertension, we analyzed the relationship between hypertension, PA level and WT indices.

In all-comers, the top 10% and 1% most physically active by total and vigorous MET-min/week and, where available, mean acceleration vector, respectively, participants were 1:1 nearest neighbour-matched without replacement based on their propensity score. We calculated the propensity score or probability that a participant had hypertension, using a GLM that adjusted for potential confounders: age, sex, ethnicity, height, BMI, smoking status, hyperlipidaemia and diabetes (see Supplementary data online, *Figure S10*). Confounding variables were selected based on their recognized association with hypertension. Inter-group differences of median volumetric, geometric parameters, *LVEF,* Global longitudinal strain (GLS), and *native T1* were assessed with the Mann–Whitney *U* test.

Data were analyzed with R version 4.4.0 (Vienna, Austria: R Core Team 2023, available at https://www.r-project.org). Important statistical packages were *tidyverse, lubridate, survminer, ggforestplot, caret, boot, MatchIt* and *DescTools*.^28–30^ We assessed statistical significance at a Bonferroni-adjusted *a priori* α-level of 0.05.

## Results

From 44 930 UKBB participants with available CMR scans, 44 438 (98.9%) with mean age 64.6 ± 7.7 years and 21 370 (48.1%) males were included (*Table 1*). After exclusion of 7358 with CVD, 37 080 participants were dichotomized into healthy reference (52.7%) and CVRF (47.3%) cohorts, respectively (see Supplementary data online, *Figure S11*).

**Table 1:** Baseline clinical and CMR-derived characteristics

	Total cohort (*n* = 44 438)	Healthy reference (*n* = 19 531)	CV risk factors (*n* = 17 549)	*P*-value
Age (years)	64.6 ± 7.7	62.6 ± 7.5	65.6 ± 7.6	<0.001
Male	21 370 (48.1)	7790 (39.9)	9081 (51.7)	<0.001
White (vs. non-White)	43 167 (97.1)	19 050 (97.5)	16 949 (96.6)	<0.001
Current smoker	1537 (3.5)	0 (0.0)	1273 (7.3)	<0.001
Height (cm)	170 ± 9.4	170 ± 9.3	170 ± 9.5	0.002
BMI (kg/m^2^)	26.5 ± 4.4	24.5 ± 2.8	28.3 ± 4.8	<0.001
Mean acceleration vector (milli-gravity)^b^	27.6 (23.0, 32.9)	29.4 (24.7, 35.0)	26.4 (22.1, 31.4)	<0.001
Total MET-min/week	2000 (1,076, 3507)	2131 (1,207, 3651)	1864 (980, 3349)	<0.001
Total moderate-vigorous MET-min/week	1120 (473, 2220)	1240 (540, 2340)	1020 (400, 2100)	<0.001
Cardiovascular risk factors				
Obesity (BMI ≥ 30.0 kg/m^2^)	8004 (18.0)	—	6310 (36.0)	—
Hypertension	12 156 (27.4)	—	8215 (46.8)	—
Hyperlipidaemia	14 141 (31.8)	—	9826 (56.0)	—
Diabetes	2696 (6.1)	—	1836 (10.5)	—
CVD				
Coronary artery disease	2601 (5.9)	—	—	—
Chronic kidney disease	3090 (7.0)	—	—	—
Cerebrovascular disease	720 (1.6)	—	—	—
Peripheral vascular disease	255 (0.57)	—	—	—
Other cardiac disease				
AF	1359 (3.1)	—	—	—
Aortic stenosis	88 (0.20)	—	—	—
Mitral valve disease	171 (0.38)	—	—	—
HCM	30 (0.07)	—	—	—
HF	306 (0.69)	—	—	—
CMR dimensional indices				
Mean absolute deviation of max. segmental wall thickness (MadWT, mm)	0.94 (0.76, 1.15)	0.86 (0.70, 1.1)	1.0 (0.81, 1.2)	<0.001
Maximum end-diastolic wall thickness (MaxWT, mm)	9.28 (8.24, 10.37)	8.8 (7.8, 9.7)	9.6 (8.7, 10.7)	<0.001
Mean end-diastolic wall thickness (MeanWT, mm)	7.1 (6.4, 7.8)	6.7 (6.1, 7.4)	7.3 (6.7, 8.1)	<0.001
CMR volumes and mass				
LVMi (g/m^2^)	44.7 (39.5, 51.0)	43.2 (38.3, 49.3)	45.6 (40.4, 51.8)	<0.001
LVEDVi (mL/m^2^)	77.5 (69.1, 86.8)	78.4 (70.5, 87.8)	76.0 (67.9, 85.2)	<0.001
LVMVR (g/mL)	0.57 (0.52, 0.64)	0.55 (0.51, 0.60)	0.60 (0.55, 0.66)	<0.001
LVEF (%)	59.7 (55.7, 63.7)	59.7 (56.0, 63.6)	59.9 (55.9, 63.9)	0.11
CMR strain parameters				
GLS (%)^a^	18.0 ± 2.3	18.3 ± 2.1	17.9 ± 2.2	<0.001
GCS (%)^b^	18.6 ± 2.3	18.8 ± 2.2	18.6 ± 2.3	<0.001
CMR tissue characterization				
Native T1 (ms)^a^	931 (910, 954)	934 (913, 957)	929 (908, 950)	<0.001

Data are in mean ± standard deviation (SD) or median (interquartile range [IQR]) for numerical variables and *n* (%) for categorical variables unless otherwise stated. BMI, body mass index; CMR, cardiovascular magnetic resonance imaging; CV, cardiovascular.

^a^<5% missing data.

^b^>5% missing data.

In comparison to the CVRF cohort, the healthy reference cohort was younger, more physically active by MET-min/week and accelerometry with a higher proportion of women (*Table 1*). The healthy reference cohort had lower median *MadWT* [0.86 (0.70, 1.1) mm vs. 1.0 (0.81, 1.2) mm], *MaxWT* [8.8 (7.8, 9.7) mm vs. 9.6 (8.7, 10.7) mm] and *MeanWT* [6.7 (6.1, 7.4) mm vs. 7.3 (6.7, 8.1) mm]. Median *LVMi* was lower [43.2 (38.3, 49.3) g/m^2^ vs. 45.6 (40.4, 51.8) g/m^2^] and median *LVEDVi* was greater [78.4 (70.5, 87.8) mL/m^2^ vs. 76.0 (67.9, 85.2) mL/m^2^]. The healthy reference cohort had higher mean *GLS* (18.3 ± 2.1% vs. 17.9 ± 2.2%) and Global circumferential strain (18.8 ± 2.2% vs. 18.6 ± 2.3%) vs. the CVRF cohort (*Table 1*). Average regional *native T1* were numerically comparable between the two cohorts [934 (913, 957) ms vs. 929 (908, 950) ms].

### Association with prevalent CV risk

On multivariable regression, 1 SD increase in age, height, BMI, and TPA level was associated with higher *MadWT*. Male sex, active smoking, hypertension and diabetes were also associated with higher *MadWT* (see Supplementary data online, *Table S3*). Excluding diabetes status in men, these associations remained significant when stratified by sex (see Supplementary data online, *Table S3*). Age, non-white ethnicity, and CVRF, particularly diabetes, had a greater effect size on *MadWT* in women (*P* < 0.001). Although the effect of TPA was comparable across men and women, vigorous PA was more strongly associated with higher *MadWT* in men (*P* < 0.001).

### Association with incident CVD

Of 37 080 participants with a median follow-up of 5.7 (4.9, 7.1) years, 638 (1.7%) experienced MACE, including 328 (0.9%) MIs and 296 (0.8%) strokes. HF and arrhythmia events were diagnosed in 232 (0.6%) and 920 (2.5%) participants, respectively. Death occurred in 554 (1.5%) participants, of which 80 (14.4%) were considered CV deaths.

#### Biomarker predictive strength

In multivariable regression models adjusting for CVRF (Model 2), 1 SD increase in *MadWT, MaxWT*, *MeanWT*, and *LVMi* was associated with 28% [HR 1.28 (1.19–1.38)], 38% [HR 1.38 (1.26–1.51)], 54% [HR 1.54 (1.40–1.70)], and 39% [HR 1.39 (1.30–1.50)] greater risk of MACE respectively (see Supplementary data online, *Figure S12* and *Table S4*), including MI, stroke and CV death (see Supplementary data online, *Table S5* and *Figure S13*). One SD increase in *MadWT, MaxWT*, *MeanWT*, and *LVMi* was associated with 30% [HR 1.30 (1.16–1.46)], 51% [HR 1.51 (1.31–1.74)], 79% [HR 1.79 (1.55–2.08)], and 100% [HR 2.00 (1.83–2.19)] greater risk of HF respectively; and 33% [HR 1.33 (1.25–1.41)], 48% [HR 1.48 (1.38–1.60)], 65% [HR 1.65 (1.53–1.79)], and 51% [HR 1.51 (1.42–1.60)] greater risk of arrhythmia respectively, (see Supplementary data online, *Figure S12* and *Table S4*).

There was a trend to stronger associations between WT indices and incident HF and arrhythmia events in men vs. women, which was not observed in the modelling of future MACE events separately (see Supplementary data online, *Table S6* and *Figure S14*). The WT indices were not predictive of death from any cause in the overall cohort and when stratified by sex (*P* < 0.05).

#### Added prognostic value of biomarker

After adjusting for clinically integrated CMR biomarkers in Model 3, the effect size magnitude and independence of WT indices to predict MACE, HF, and arrhythmia were maintained (see Supplementary data online, *Figure S12* and *Table S4*). *MadWT, MaxWT*, *MeanWT*, and *LVMi* provided incremental prognostic value above *LVEDVi*, *LVEF*, and CVRF to predict said events: MACE △C-index 0.007, 0.008, 0.009, and 0.012 respectively (*P* < 0.001, for all); HF △C-index 0.003, 0.003, 0.003, and 0.002 respectively (*P* < 0.05 for all); and arrhythmia △C-index 0.009, 0.012, 0.014, and 0.005 respectively (*P* < 0.001 for all) (see Supplementary data online, *Table S4*).

#### Significance of LVWT beyond LVM

**Figure 2:**
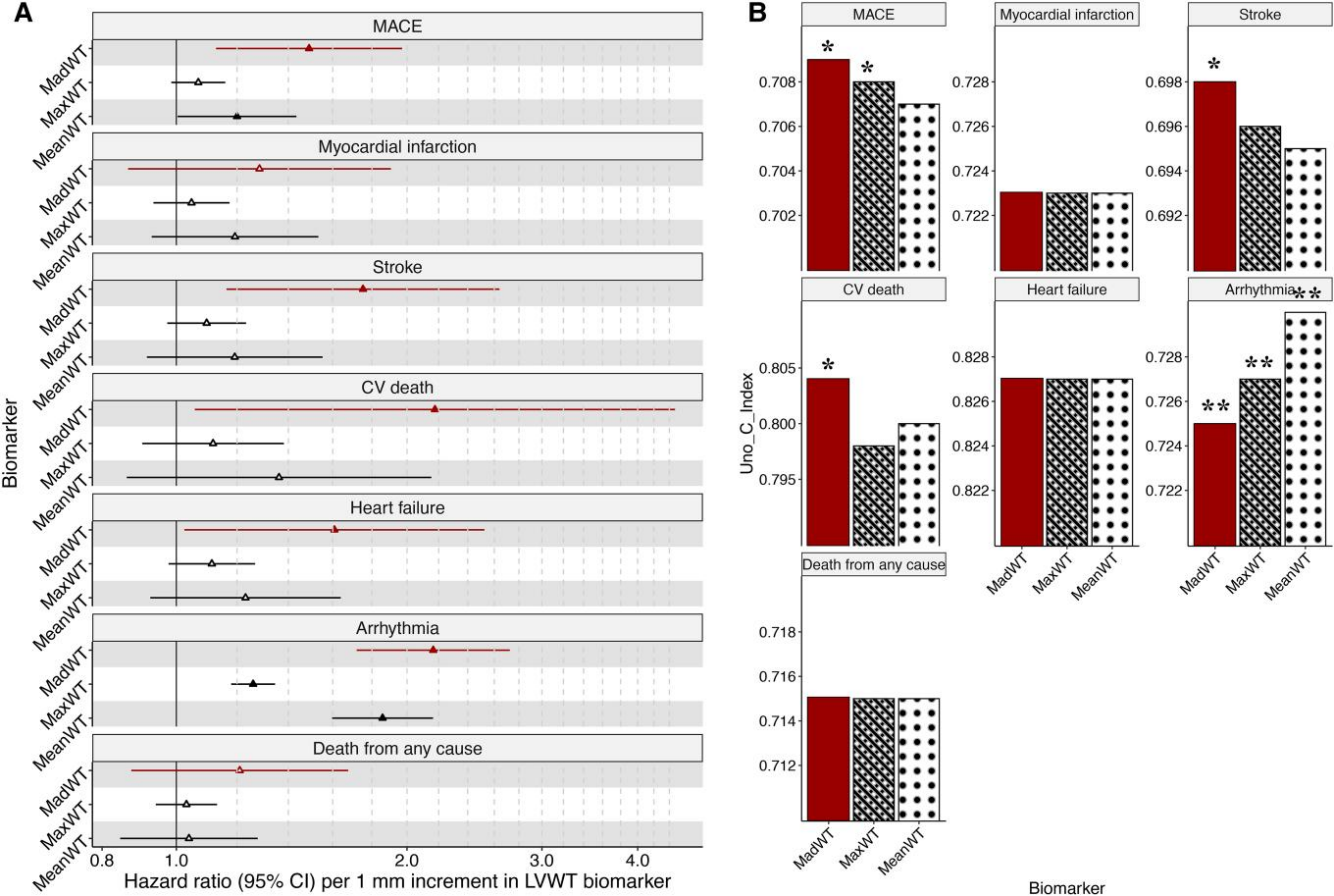
Forest plot of effect sizes (*A*) and bar charts of C indices (*B*), comparing LVWT indices to predict CV endpoints. After adjusting for CV risk factors and established CMR biomarkers in Model 4, all three LVWT indices were strongly associated with arrhythmia events, but only *MadWT* remained predictive of MACE, HF, stroke and CV death (*P* < 0.05). Accordingly, the overall discriminative ability of Model 4 for arrhythmia was improved by *MadWT*, *MaxWT*, and *MeanWT*, for MACE by *MadWT* and *MaxWT* and for stroke and CV death only *MadWT* (△C-index *P* < 0.05). ^†^Filled vs. unfilled marker indicates *P* < 0.05 vs. ≥0.05. CI, confidence interval; CMR, cardiovascular magnetic resonance imaging; CV, cardiovascular; HF, heart failure; LVWT, left ventricular wall thickness; MACE, major adverse cardiovascular event; MadWT, mean absolute deviation of maximum segmental wall thickness; MaxWT, maximum end-diastolic wall thickness; MeanWT, mean end-diastolic wall thickness.

After adjusting for *LVMi* in the exploratory Model 4, a 1 mm increase in *MadWT* was associated with greater risk of HF by 61% [HR 1.61 (1.02–2.52)]; arrhythmia by 116% [HR 2.16 (1.72–2.72)]; and MACE by 49% [HR 1.49 (1.13–1.97)], driven by its association with stroke [HR 1.75 (1.16–2.64)] and CV death [HR 2.18 (1.06–4.48)]. By comparison, a 1 mm increase in *MeanWT* and *MaxWT* was only associated with greater risk of arrhythmia events [HR 1.86 (1.60–2.16)] and [HR 1.26 (1.18–1.35)] respectively. *MeanWT* was borderline significantly associated with MACE [HR 1.20 (1.00–1.43 per 1 mm △*MeanWT*)] (*Figure 2*, Supplementary data online, *Tables S4* and *S5*). Overall model discrimination was improved for arrhythmia by *MadWT, MaxWT*, and *MeanWT* [△C-index 0.005, 0.007, and 0.011 respectively (*P* < 0.001 for all)], for MACE by *MadWT* and *MaxWT* [△C-index 0.001 for both respectively (*P* = 0.003)], and for stroke and CV death only by *MadWT* [△C-index 0.002 (*P* = 0.002) and 0.004 (*P* = 0.001), respectively] (*Figure 2*, Supplementary data online, *Tables S4* and *S5*).

### Hypertension, exercise and LVWT

**Figure 3:**
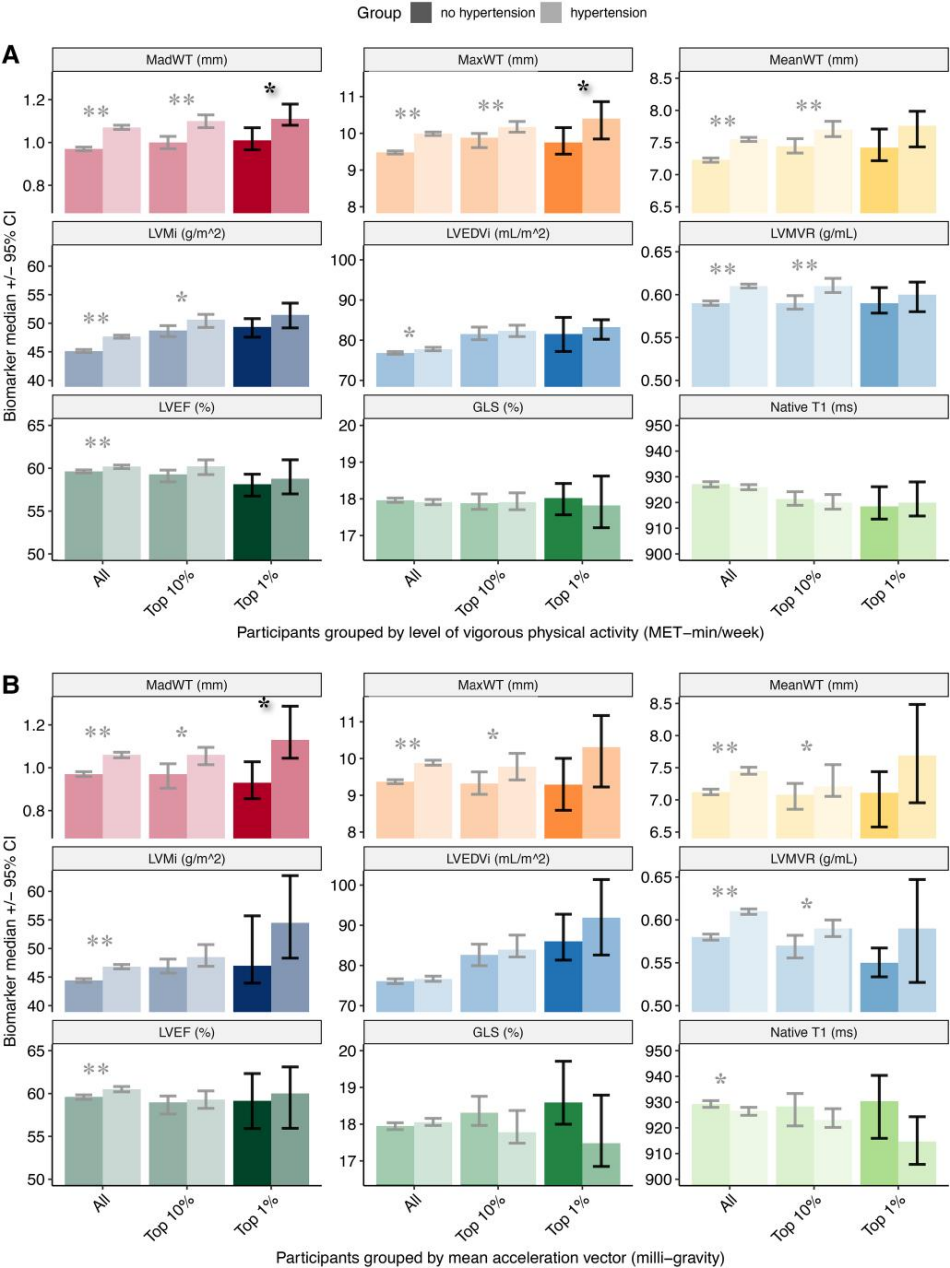
Grouped bar charts, comparing CMR-derived WT, volume and functional parameters by hypertension status and PA level. In PSM cohorts, WT indices *MadWT, MaxWT*, *MeanWT* and volumetric parameters *LVEDVi, LVMi, LVMVR, LVEF*, but not *GLS* and *native T1* were significantly different between hypertensive and non-hypertensive participants. In the top 1% most physically active by vigorous MET-min/week (Panel *A*) as well as mean acceleration vector (Panel *B*), only *MadWT* was consistently at least nominally significantly different between hypertensive and non-hypertensive groups with no overlap of their 95% CIs. *Bonferroni-corrected *P* < 0.006. **Bonferroni-corrected *P* < 0.0001. CI, confidence interval; CMR, cardiovascular magnetic resonance imaging; GLS, global longitudinal strain; LVEDVi, indexed left ventricular end-diastolic volume; LVEF, left ventricular ejection fraction; LVMi, indexed left ventricular mass; LVMVR, left ventricular mass to volume ratio; MadWT, mean absolute deviation of maximum segmental wall thickness; MaxWT, maximum end-diastolic wall thickness; MeanWT, mean end-diastolic wall thickness; MET, metabolic equivalent of task; PA, physical activity; WT, wall thickness.

In PSM cohorts of hypertensive and non-hypertensive participants, *n* = 16 430 with self-reported MET-min/week and *n* = 6794 with accelerometry data, *MadWT, MaxWT*, *MeanWT*, *LVMi*, and *LVMVR,* but not *LVEDVi, LVEF*, *GLS*, and *native T1* were consistently significantly different between hypertensive and non-hypertensive participants (*P* < 0.05 vs. *P* > 0.05 respectively) (see Supplementary data online, *Tables S7*–*S9*). In the top 1% most physically active by total, vigorous MET-min/week and mean acceleration vector, *MadWT* was significantly different between hypertensive and non-hypertensive groups (*P* < 0.05) with no overlap of their 95% CIs [median 1.09 (1.01–1.13) mm vs. median 0.96 (0.89–1.00) mm; median 1.11 (1.08–1.18) mm vs. median 1.01 (0.96–1.06) mm; and median 1.13 (1.04–1.29) mm vs. median 0.93 (0.86–1.03) mm respectively] (*Figure 3* and Supplementary data online, *Tables S7*–*S9*). Of the remaining investigated biomarkers including *native T1*, *LVMVR* by total MET-min/week, and *MaxWT* by vigorous MET-min/week, were borderline significantly different between hypertensive and non-hypertensive groups in the top 1% most physically active (*Figure 3* and Supplementary data online, *Tables S7*–*S9*). *MadWT* had the greatest relative difference between hypertension and no hypertension across the 3 PSM cohorts stratified by total and vigorous MET-min/week and mean acceleration vector (see Supplementary data online, *Figure S15*).

## Discussion

We present a novel CMR-based population study of a LVWT heterogeneity biomarker, *MadWT* (Central Illustration). Our major findings are as follows: (i) Common CVRF are associated with greater *MadWT*; (ii) *MadWT* provides independent incremental prognostic value above standard CMR-derived biomarkers to predict MACE and HF, outperforming *MaxWT* and *MeanWT* and finally, (iii) *MadWT* consistently distinguished hypertensives and non-hypertensives in the top 1% most physically active participants.

Our results add to the expanding evidence base that increased LVM and LVWT confer greater risk of HF and MACE.^1,2,31–33^ In two population sub-studies free of clinical CVD, the Framingham Heart Study (*n* = 1715) and MESA (*n* = 4988), elevated *LVMi* measured by CMR conferred greater risk of adverse CV events independent of traditional risk factors and coronary artery calcium score.^32,33^ Lundin and colleagues then calculated global wall thickness (GT) from CMR-derived *LVMi* and *LVEDVi*, akin to our *MeanWT*. They demonstrated that patients with concentric remodelling, defined by an elevated GT, but normal *LVMi, LVEDVi*, and *LVEF* were at greater risk of HF and all-cause death than patients with normal indices of cardiac structure and function.^31^ Our observation of an association between *MeanWT* and incident MACE, but not HF, after adjusting for *LVMi,* lends weight to the assertion by Bluemke *et al.*^1^ that concentric remodelling and hypertrophy may be more predictive of ischaemic and HF events, respectively.^1^ Mass and WT measure different components of LVH; both should be routinely assessed on cardiac imaging.

An important finding is that *MadWT* provided independent and incremental prognostic value to predict future MACE, HF and arrhythmia beyond CVRF and established CMR biomarkers, particularly left ventricular mass. Whereas *MeanWT* and *LVMi* measure totality of LVWT, *MadWT* and to a lesser degree *MaxWT* describe LVWT heterogeneity.^16^ Previously, de Marvao *et al.*^34^ used 3-dimensional CMR to show regionality in the hypertrophic response to hypertension. Yuan *et al.*^5^ demonstrated that septal and apical hypertrophy provided separate additive risk for future cardiomyopathy and AF above total LVM.^5,34^  *MadWT* has the advantage of quantifying LVWT heterogeneity across the entire ventricle.

Furthermore, differentiating physiological from pathological LVH remains a conundrum, especially in young competitive athletes with mild LVH (13 mm ≤ LVWT > 15 mm).^8–10,12,13^ Existing studies are limited by sample size and methodology; small cohorts of athletes in screening programs were previously compared with sedentary patients with LVH phenotypes.^8–10,12,13^ In contrast, we leveraged a UKBB CMR cohort of >35 000 without clinical CVD to characterize the relationship between hypertension, a common cause of LVH, and CMR-derived volume, dimension, functional indices and *native T1* tissue characterisation across different levels of PA. We found that *MadWT* was consistently different between hypertensive and non-hypertensive in the top 1% most physically active by self-reported questionnaires and accelerometry. Although *LVMVR* by total MET-min/week and *MaxWT* by vigorous MET-min/week were different between said groups, findings were not reproduced after objectively stratifying PA level by accelerometry. Acknowledging the limitations of an indirect comparison of a community cohort, these results corroborate our hypothesis that LVWT heterogeneity could differentiate uniform physiological LVH from pathological LVH (*Graphical Abstract*).

### Strengths and limitations

The strength of our study is derived from the quality and size of the data collected and the availability of PA and longitudinal data.

An important limitation is our use of a predominantly White, at least middle-aged, community-based cohort. It is vitally important that *MadWT* is investigated in other ethnicities, particularly African/Afro-Caribbean, who are known to exhibit greater hypertrophic responses to exercise.^35,36^ Although this impacts the generalisability of our findings, we postulate a greater divergence in *MadWT* between younger healthy athletes and athletes with an early HCM phenotype. Despite the unavailability of LGE, an established discriminator between pathological and physiological LVH, we demonstrated the superiority of *MadWT* against highly sensitive *GLS* and *native T1* tissue characterisation. The use of questionnaire-based quantification of PA level also introduces significant recall bias; however, replicability with objective accelerometer data in an albeit smaller cohort strengthens the reliability of our results. Finally, our study’s retrospective observational design means confounding and reverse causation cannot be excluded. This is particularly relevant to our propensity score matching methodology. Although balance plots demonstrated well-matched groups, we did not adjust for co-morbidities, medications as well as unrecognized confounding factors.

### Clinical perspectives

In the era of growing health data-minded populations and automated medical image analysis, we envision that the machine learning non-contrast derived biomarker, *MadWT,* will become a CMR reporting standard, particularly in the setting of unexplained LVH. Integration of deep learning-assisted segmentation means that segmental WT and *MadWT* are rapidly and reproducibly computable on standard bSSFP cine sequences. As a novel imaging-derived phenotype, *MadWT* may identify to date unrecognized susceptibility genes for HCM and improve ECG-predicted LVH phenotypes by test of association, facilitating more sensitive ECG-based sports preparticipation screening.^37,38^

## Conclusion

Assessment of LVWT heterogeneity is clinically relevant for prognosis and may facilitate diagnosis in physically active populations with unexplained LVH. To our knowledge, this is the first study to describe a novel LVWT heterogeneity biomarker, *MadWT* that adds prognostic value above left ventricular mass to risk-stratify incident ischaemic and HF events in a low-risk general population. At the same time, *MadWT* may discriminate between very physically active hypertensive and non-hypertensive populations better than conventional CMR dimension, volume, and functional parameters. Our study sets the stage for further investigations into *MadWT* in other ethnicities, athlete, and disease cohorts. We are optimistic that *MadWT* will be imminently part of deep learning-assisted CMR reporting protocols for suspected cardiomyopathy, especially in young physically active individuals.

## Supplementary Material

qyaf092_Supplementary_Data

## Data Availability

This research was conducted using the UK Biobank resource under access application 2964. UK Biobank will make the data available to all bona fide researchers for all types of health-related research that is in the public interest, without preferential or exclusive access for any persons. All researchers will be subject to the same application process and approval criteria as specified by UK Biobank. For more details on the access procedure, see the UK Biobank website: http://www.ukbiobank.ac.uk/register-apply/.

## Lead author biography

Dr Steffen Petersen is a Professor of Cardiovascular Medicine at Queen Mary University of London and a Consultant Cardiologist at Barts Heart Centre, St Bartholomew’s Hospital in London. His many roles include Deputy Director of the British Heart Foundation Data Science Centre as well as previous President of the European Association of Cardiovascular Imaging. His research focuses on cardiac imaging, particularly CMR imaging and its application in epidemiological studies as well as integration with artificial intelligence solutions in healthcare. He has successfully supervised more than 15 PhD students since 2006 all of whom completed on time.

Dr Nay Aung is a NIHR Academic Clinical Lecturer in Omics and Bioinformatics at the William Harvey Research Institute as well as a Consultant Cardiologist at Barts Heart Centre, St Bartholomew’s Hospital in London. Clinically, Dr Aung Dr Aung specializes in heart failure, inherited cardiac conditions, and multi-modal cardiac imaging. Academically, Dr Aung combines artificial intelligence and machine learning with genomics and cardiac imaging to conduct novel cardiovascular epidemiological studies.
