# Supplementary material for: Left ventricular wall thickness heterogeneity improves cardiovascular disease diagnosis and prognosis: a UK Biobank cardiovascular magnetic resonance cohort study
Source: Eur Heart J Imaging Methods Pract. 2025 Jul 18;3(2):qyaf092. doi: 10.1093/ehjimp/qyaf092 (PMC12308483; doi:10.1093/ehjimp/qyaf092)
Supplement: qyaf092_Supplementary_Data [file qyaf092_supplementary_data.docx]

**SUPPLEMENTAL APPENDIX**

**Table of Contents**

[Table S1. Definitions of important disease phenotypes based on variables and outcomes available in the UK Biobank. 2](#_Toc203632441)

[Table S2. Variance inflation factor of predictor variables in regression models 2 and 3 by endpoint and biomarker. 16](#_Toc203632442)

[Table S3. Multivariable regression models, demonstrating the relationship between CV risk factors, physical activity and *MadWT*. 21](#_Toc203632443)

[Table S4. Results of Cox proportional hazards regression models to predict primary study endpoints by WT indices. 22](#_Toc203632444)

[Table S5. Results of Cox proportional hazards regression models to predict myocardial infarction, stroke and CV death by WT indices. 26](#_Toc203632445)

[Table S6. Cox proportional hazards regression models to predict major study endpoints by WT indices in men and women. 29](#_Toc203632446)

[Table S7. WT & volumetric parameters in PSM hypertensive vs. non-hypertensive cohorts, stratified by total PA (MET-min/week). 32](#_Toc203632447)

[Table S8. WT & volumetric parameters in PSM hypertensive vs. non-hypertensive cohorts, stratified by vigorous PA (MET-min/week). 35](#_Toc203632448)

[Table S9: WT & volumetric parameters in PSM HTN vs. non-HTN cohorts, stratified by mean acceleration vector (milli-gravity). 38](#_Toc203632449)

[Figure S1. *MadWT* distribution, stratified by number of measured LV segments. 41](#_Toc203632450)

[Figure S2. Density (A) and QQ (B) plots of CMR-derived biomarkers. 42](#_Toc203632451)

[Figure S3. Incident MACE covariate residual *vs.* time plots. 43](#_Toc203632452)

[Figure S4. Incident heart failure covariate residual *vs.* time plots. 44](#_Toc203632453)

[Figure S7. Incident myocardial infarction covariate residual *vs.* time plots. 47](#_Toc203632454)

[Figure S9. CV death covariate residual *vs.* time plots. 49](#_Toc203632455)

[Figure S10: Balance plots for PSM hypertensive and non-hypertensive cohorts. 50](#_Toc203632456)

[Figure S11. Study flowchart. 51](#_Toc203632457)

[Figure S12. Forest plot, comparing WT indices to predict CV endpoints. 52](#_Toc203632458)

[Figure S13. Forest plot of WT indices & *LVMi* to predict MACE sub-endpoints. 53](#_Toc203632459)

[Figure S14. Forest plot of WT indices and *LVMi* to predict CV endpoints by sex. 54](#_Toc203632460)

[Figure S15. Relative difference of CMR-derived parameters by HTN status & PA level. 55](#_Toc203632461)

**Tables**

# Table S1. Definitions of important disease phenotypes based on variables and outcomes available in the UK Biobank.

| **Phenotype** | **Data fields** | **Field names** | **Data codes** | **Data code definitions** |
| --- | --- | --- | --- | --- |
| Aortic stenosis | 20002 | Non-cancer illness code, self-reported | 1490 | Aortic stenosis |
|  | 41202  41204  41270  40001  40002 | Diagnoses – main ICD10  Diagnoses – secondary ICD10  Diagnoses – ICD10  Underlying (primary) cause of death: ICD10  Contributory (secondary) cause of death: ICD10 | I06.0, I06.2, I35.0, I35.2 | Rheumatic aortic stenosis, Rheumatic aortic stenosis with insufficiency, Aortic (valve) stenosis, Aortic (valve) stenosis with insufficiency |
|  | 41200  41210  41272 | Operative procedures – main OPCS4  Operative procedures – secondary OPCS4  Operative procedures - OPCS4 | K35.2 | Percutaneous transluminal aortic valvotomy |
|  | 41256  41258  41273 | Operative procedures – main OPCS3  Operative procedures – secondary OPCS3  Operative procedures – OPCS3 | 314.2 | Other valvuloplasty: aortic valve |
| Atrial fibrillation  or flutter | 20002 | Non-cancer illness code, self-reported | 1471, 1483 | Atrial fibrillation, Atrial flutter |
|  | 41202  41204  41270  40001  40002 | Diagnoses – main ICD10  Diagnoses – secondary ICD10  Diagnoses – ICD10  Underlying (primary) cause of death: ICD10  Contributory (secondary) cause of death: ICD10 | I48, I48.0, I48.1, I48.2, I48.3, I48.4, I48.9 | Atrial fibrillation and flutter, Paroxysmal atrial fibrillation, Persistent atrial fibrillation, Chronic atrial fibrillation, Typical atrial flutter, Atypical atrial flutter, Atrial fibrillation and atrial flutter, unspecified |
|  | 41203  41205  41271 | Diagnoses – main ICD9  Diagnoses – secondary ICD9  Diagnoses – ICD9 | 4273 | Atrial fibrillation and flutter |
|  | 41200  41210  41272 | Operative procedures – main OPCS4  Operative procedures – secondary OPCS4  Operative procedures - OPCS4 | K57.1, K62.1, K62.2, K62.3, K62.4 | Percutaneous transluminal ablation of atrioventricular node, Percutaneous transluminal ablation of pulmonary vein to left atrium conducting system, Percutaneous transluminal ablation of atrial wall for atrial flutter, Percutaneous transluminal ablation of conducting system of heart for atrial flutter NEC, Percutaneous transluminal internal cardioversion NEC |
| Bradyarrhythmia | 20002 | Non-cancer illness code, self-reported | 1486 | Sick sinus syndrome |
|  | 20004 | Operation code, self-reported | 1548 | Pacemaker insertion |
|  | 41202  41204  41270  40001  40002 | Diagnoses – main ICD10  Diagnoses – secondary ICD10  Diagnoses – ICD10  Underlying (primary) cause of death: ICD10  Contributory (secondary) cause of death: ICD10 | I44, I44.1, I44.2, I44.3, I44.5, I49.5 | Atrioventricular and left bundle-branch block, Atrioventricular block, second degree, Atrioventricular block, complete, Other and unspecified atrioventricular block, Other specified heart block, Sick sinus syndrome |
|  | 41203  41205  41271 | Diagnoses – main ICD9  Diagnoses – secondary ICD9  Diagnoses – ICD9 | 4260, 4261, 4266 | Atrioventricular block, complete, Atrioventricular block, other and unspecified, Other specified heart block |
|  | 41200  41210  41272 | Operative procedures – main OPCS4  Operative procedures – secondary OPCS4  Operative procedures - OPCS4 | K60, K60.1, K60.2, K60.3, K60.4, K60.5, K60.6, K60.8, K60.9, K61, K61.1, K61.2,  K61.3, K61.4, K61.5, K61.6, K61.8, K61.9  K73, K73.1, K73.2, K73.3, K73.8, K73.9, K74, K74.1, K74.2, K74.8, K74.9 | Cardiac pacemaker system introduced through vein, Implantation of intravenous cardiac pacemaker system NEC, Resiting of lead of intravenous cardiac pacemaker system, Renewal of intravenous cardiac pacemaker system, Removal of intravenous cardiac pacemaker system, Implantation of intravenous single chamber cardiac pacemaker system, Implantation of intravenous dual chamber cardiac pacemaker system, Other specified cardiac pacemaker system introduced through vein, Unspecified cardiac pacemaker system introduced through vein, Other cardiac pacemaker system, Implantation of cardiac pacemaker system NEC, Resiting of lead of cardiac pacemaker system NEC, Renewal of cardiac pacemaker system NEC, Removal of cardiac pacemaker system NEC, Implantation of single chamber cardiac pacemaker system, Implantation of dual chamber cardiac pacemaker system, Other specified other cardiac pacemaker system, Unspecified other cardiac pacemaker system,  Other cardiac pacemaker system introduced through vein, Renewal of intravenous single chamber cardiac pacemaker system, Renewal of intravenous dual chamber cardiac pacemaker system, Other specified other cardiac pacemaker system introduced through vein, Unspecified other cardiac pacemaker system introduced through vein, Cardiac pacemaker system, Renewal of single chamber cardiac pacemaker system NEC, Renewal of dual chamber cardiac pacemaker system NEC, Other specified cardiac pacemaker system, Unspecified cardiac pacemaker system |
|  | 41256  41258  41273 | Operative procedures – main OPCS3  Operative procedures – secondary OPCS3  Operative procedures – OPCS3 | 305 | Insertion of pace-maker |
| Cerebrovascular disease/Stroke | 20002 | Non-cancer illness code, self-reported | 1081, 1082, 1491, 1986 | Stroke, Transient ischaemic attack (tia), Brain haemorrhage, Subarachnoid haemorrhage, |
|  | 20004 | Operation code, self-reported | 1105, 1106, 1109 | Carotid artery surgery/endarterectomy, Cerebral artery aneurysm surgery or clipping, Carotid artery angioplasty +/- stent |
|  | 41202  41204  41270  40001  40002 | Diagnoses – main ICD10  Diagnoses – secondary ICD10  Diagnoses – ICD10  Underlying (primary) cause of death: ICD10  Contributory (secondary) cause of death: ICD10 | G46, I60, I61, I62, I63, I64 | Vascular syndromes of brain in cerebrovascular diseases, Subarachnoid haemorrhage, Intracerebral haemorrhage, Other nontraumatic intracranial haemorrhage, Cerebral infarction, Stroke not specified as haemorrhage or infarction |
|  | 41203  41205  41271 | Diagnoses – main ICD9  Diagnoses – secondary ICD9  Diagnoses – ICD9 | 430, 431, 433, 434, 436 | Subarachnoid haemorrhage, Intracerebral haemorrhage, Occlusion and stenosis of precerebral arteries, Occlusion of cerebral arteries, Acute but ill-defined cerebrovascular disease |
|  | 41200  41210  41272 | Operative procedures – main OPCS4  Operative procedures – secondary OPCS4  Operative procedures - OPCS4 | V03.7 | Decompressive craniectomy |
| Chronic kidney disease | 20002 | Non-cancer illness code, self-reported | 1192, 1193, 1194, 1196, 1197, 1200, 1405, 1427, 1519, 1520, 1607, 1608, 1609 | Renal/kidney failure, Renal failure requiring dialysis, Renal failure not requiring dialysis, Urinary tract infection/kidney infection, Kidney stone/ureter stone/bladder stone, Ureteric obstruction/hydronephrosis, Other renal/kidney problem, Polycystic kidney, Kidney nephropathy, Iga nephropathy, Diabetic nephropathy, Nephritis, Glomerulonephritis, |
|  | 20004 | Operation code, self-reported | 1195, 1197, 1487, 1580, 1581, 1582, 1618 | Renal/kidney transplant, Percutaneous/open kidney stone surgery/lithotripsy, Nephrectomy/kidney removed, Dialysis access surgery, Haemodialysis access / fistula surgery, Peritoneal dialysis (capd) access surgery, Renal biopsy/kidney biopsy |
|  | 41202  41204  41270  40001  40002 | Diagnoses – main ICD10  Diagnoses – secondary ICD10  Diagnoses – ICD10  Underlying (primary) cause of death: ICD10  Contributory (secondary) cause of death: ICD10 | I12, I13, N00,  N01, N02, N03, N04, N05, N06, N07, N08, N10, N11, N12, N13, N14, N15, N16, N17. N18, N19, N20, N21, N22  N23, N25, N26, N27, N28, N29, N31, C64, C65 | Hypertensive renal disease, Hypertensive heart and renal disease, Acute nephritic syndrome, Rapidly progressive nephritic syndrome, Recurrent and persistent haematuria, Chronic nephritic syndrome, Nephrotic syndrome, Unspecified nephritic syndrome, Isolated proteinuria with specified morphological lesion, Hereditary nephropathy, not elsewhere specified, Glomerular disorders in diseases classified elsewhere, Acute tubulo-interstitial nephritis, Chronic tubule-interstitial nephritis, Tubulo-interstitial nephritis, not specified as acute or chronic, Obstructive and reflux uropathy, Drug- and heavy-metal-induced tubule-interstitial and tubular conditions, Other renal tubule-interstitial diseases, Renal tubule-interstitial disorders in disease classified elsewhere, Acute renal failure, Chronic renal failure, Unspecified renal failure, Calculus of kidney and ureter, Calculus of lower urinary tract, Calculus of urinary tract in diseases classified elsewhere, Unspecified renal colic, Disorders resulting from impaired renal tubular function, Unspecified contracted kidney, Small kidney of unknown cause, Other disorders of kidney and ureter, not elsewhere classified, Other disorders of kidney and ureter in diseases classified elsewhere, Neuromuscular dysfunction of bladder, not elsewhere classified, Malignant neoplasm of kidney, except renal pelvis, Malignant neoplasm of renal pelvis |
|  | 41203  41205  41271 | Diagnoses – main ICD9  Diagnoses – secondary ICD9  Diagnoses – ICD9 | 189, 403, 581, 582, 583, 584, 585, 586, 587, 588, 589, 590, 591, 592, 593 | Malignant neoplasm of kidney and other and unspecified urinary organs, Hypertensive renal disease, Nephrotic syndrome, Chronic glomerulonephritis, Nephritis and nephropathy, not specified as acute or chronic, Acute renal failure, Chronic renal failure, Renal failure, unspecified, Renal sclerosis, unspecified, Disorders resulting from impaired renal function, Small kidney of unknown cause, Infections of kidney, Hydronephrosis, Calculus of kidney and ureter, Other disorders of kidney and ureter |
|  | 41200  41210  41272 | Operative procedures – main OPCS4  Operative procedures – secondary OPCS4  Operative procedures - OPCS4 | M01, M02, M03, M04, M05, M06, M08, M09, M10, M11, M13, M14, M15, M16, M17 | Transplantation of kidney, Total excision of kidney, Partial excision of kidney, Open extirpation of lesion of kidney, Open repair of kidney, Incision of kidney, Other open operations on kidney, Therapeutic endoscopic operations on calculus of kidney, Other therapeutic endoscopic operations on kidney, Diagnostic endoscopic examination of kidney, Percutaneous puncture of kidney, Extracorporeal fragmentation of calculus of kidney, Operations on kidney along nephrostomy tube track, Other operations on kidney, Interventions associated with transplantation of kidney |
|  | 41256  41258  41273 | Operative procedures – main OPCS3  Operative procedures – secondary OPCS3  Operative procedures – OPCS3 | 560, 561, 562, 563, 564, 565, 566, 567, 568, 569, 571 | Nephrotomy, not elsewhere classified : biopsy, not elsewhere classified, Nephrostomy and pyelostomy, Pyelostomy, Removal of renal calculus, Percutaneous puncture of kidney, Removal of kidney, complete, Transplantation of kidney, Excision or destruction of lesion of kidney, Repair of kidney and renal pelvis, Nephropexy, Renal arterial and venous puncture |
| Coronary artery disease | 20002 | Non-cancer illness code, self-reported | 1075 | Heart attack/myocardial infarction |
|  | 20004 | Operation code, self-reported | 1070, 1095 | Coronary angioplasty (ptca) +/- stent, Coronary artery bypass grafts (cabg) |
|  | 41202  41204  41270  40001  40002 | Diagnoses – main ICD10  Diagnoses – secondary ICD10  Diagnoses – ICD10  Underlying (primary) cause of death: ICD10  Contributory (secondary) cause of death: ICD10 | I20, I21, I22, I23, I24, I25 | Angina pectoris, Acute myocardial infarction, Subsequent myocardial infarction, Certain current complications following acute myocardial infarction, Other acute ischaemic heart diseases, Chronic ischaemic heart disease |
|  | 41203  41205  41271 | Diagnoses – main ICD9  Diagnoses – secondary ICD9  Diagnoses – ICD9 | 410, 411, 412, 413, 414 | Acute myocardial infarction, Other acute and subacute forms of ischaemic heart disease, Old myocardial infarction, Angina pectoris, Other forms of chronic ischaemic heart disease |
|  | 41200  41210  41272 | Operative procedures – main OPCS4  Operative procedures – secondary OPCS4  Operative procedures - OPCS4 | K40, K41, K42, K43, K44, K45, K46, K47, K49, K50, K51, K75 | Saphenous vein graft replacement of coronary artery, Other autograft replacement of coronary artery, Allograft replacement of coronary artery, Prosthetic replacement of coronary artery, Other replacement of coronary artery, Connection of thoracic artery to coronary artery, Other bypass of coronary artery, Repair of coronary artery, Transluminal balloon angioplasty of coronary artery, Other therapeutic transluminal operations on coronary artery, Diagnostic transluminal operations on coronary artery, Percutaneous transluminal balloon angioplasty and insertion of stent into coronary artery |
|  | 41256  41258  41273 | Operative procedures – main OPCS3  Operative procedures – secondary OPCS3  Operative procedures – OPCS3 | 304.1, 304.2, 304.3 | Operations affecting myocardium : coronary endarterectomy, Operations affecting myocardium : revascularization (poudrage), Operations affecting myocardium : coronary anastomosis or graft |
| Diabetes | 20002 | Non-cancer illness code, self-reported | 1220, 1222, 1223 | Diabetes, Type 1 diabetes, Type 2 diabetes |
|  | 41202  41204  40001  40002 | Diagnoses – main ICD10  Diagnoses – secondary ICD10  Underlying (primary) cause of death: ICD10  Contributory (secondary) cause of death: ICD10 | E10, E11, E12, E13, E14 | Insulin-dependent diabetes mellitus, Non-insulin-dependent diabetes mellitus, Malnutrition-related diabetes mellitus, Other specified diabetes mellitus, Unspecified diabetes mellitus |
|  | 41203  41205  41271 | Diagnoses – main ICD9  Diagnoses – secondary ICD9  Diagnoses – ICD9 | 250 | Diabetes mellitus |
|  | 41200  41210  41272 | Operative procedures – main OPCS4  Operative procedures – secondary OPCS4  Operative procedures - OPCS4 | J54.2, J54.4 | Transplantation of whole pancreas, Transplantation of islet of Langerhans |
|  | 6153 | Medication for cholesterol, blood pressure, diabetes, or take exogenous hormones | 3 | Insulin |
|  | 6177 | Medication for cholesterol, blood pressure or diabetes | 3 | Insulin |
|  | 20003 | Treatment/medication code | 1140874744, 1140884600, 1141171646 | Gliclazide, metformin, pioglitazone |
| Heart failure | 20002 | Non-cancer illness code, self-reported | 1076 | Heart failure |
|  | 41202  41204  40001  40002 | Diagnoses – main ICD10  Diagnoses – secondary ICD10  Underlying (primary) cause of death: ICD10  Contributory (secondary) cause of death: ICD10 | I11.0, I13.0, I13.2, I.25.5, I42.0, I42.6, I42.7, I50 | Hypertensive heart disease with (congestive) heart failure, Hypertensive heart and renal disease with (congestive) heart failure, Hypertensive heart and renal disease with both (congestive) heart failure and renal failure, Ischaemic cardiomyopathy, Dilated cardiomyopathy, Alcoholic cardiomyopathy, Cardiomyopathy due to drugs and other external agents, Heart failure |
|  | 41203  41205  41271 | Diagnoses – main ICD9  Diagnoses – secondary ICD9  Diagnoses – ICD9 | 428 | Heart failure |
|  | 41200  41210  41272 | Operative procedures – main OPCS4  Operative procedures – secondary OPCS4  Operative procedures - OPCS4 | K01, K02, K54, K56, K59.6, K59.7, K60.7, K61.7, K73.3, K74.3 | Transplantation of heart and lung, Other transplantation of heart, Open heart assist operations, Transluminal heart assist operations, Implantation of cardioverter defibrillator using three electrode leads, Renewal of cardioverter defibrillator using three electrode leads,  Implantation of intravenous biventricular cardiac pacemaker system, Implantation of biventricular cardiac pacemaker system, Renewal of intravenous biventricular cardiac pacemaker, Renewal of biventricular cardiac pacemaker NEC |
|  | 41256  41258  41273 | Operative procedures – main OPCS3  Operative procedures – secondary OPCS3  Operative procedures – OPCS3 | 319.2 | Other operations on open heart : heart assist system |
| Hyperlipidemia | 20002 | Non-cancer illness code, self-reported | 1473 | High cholesterol |
|  | 41202  41204  40001  40002 | Diagnoses – main ICD10  Diagnoses – secondary ICD10  Underlying (primary) cause of death: ICD10  Contributory (secondary) cause of death: ICD10 | E78 | Disorders of lipoprotein metabolism and other lipidaemias |
|  | 41203  41205  41271 | Diagnoses – main ICD9  Diagnoses – secondary ICD9  Diagnoses – ICD9 | 2720, 2721, 2722 | Pure hypercholesterolaemia, Hyperglyceridaemia, Mixed hyperlipidaemia |
|  | 6153 | Medication for cholesterol, blood pressure, diabetes, or take exogenous hormones | 1 | Cholesterol lowering medication |
|  | 6177 | Medication for cholesterol, blood pressure or diabetes | 1 | Cholesterol lowering medication |
|  | 20003 | Treatment/medication code | 1141146234, 1141192736,  1140861954,  1140888648,  1141192410, 1140861958 | Atorvastatin, ezetimibe, fenofibrate, pravastatin, rosuvastatin, simvastatin, |
| Hypertension | 20002 | Non-cancer illness code, self-reported | 1065, 1072 | Essential hypertension, hypertension |
|  | 41202  41204  40001  40002 | Diagnoses – main ICD10  Diagnoses – secondary ICD10  Underlying (primary) cause of death: ICD10  Contributory (secondary) cause of death: ICD10 | I10, I11, I12, I13, I14, I15 | Essential (primary) hypertension, Hypertensive heart disease, Hypertensive renal disease, Hypertensive heart and renal disease, Secondary hypertension |
|  | 41203  41205  41271 | Diagnoses – main ICD9  Diagnoses – secondary ICD9  Diagnoses – ICD9 | 401, 403 | Essential hypertension, Hypertensive renal disease |
|  | 6153 | Medication for cholesterol, blood pressure, diabetes, or take exogenous hormones | 2 | Blood pressure medication |
|  | 6177 | Medication for cholesterol, blood pressure or diabetes | 2 | Blood pressure medication |
| Hypertrophic cardiomyopathy | 20002 | Non-cancer illness code, self-reported | 1588 | Hypertrophic cardiomyopathy (hcm/hocm) |
|  | 41202  41204  41270  40001  40002 | Diagnoses – main ICD10  Diagnoses – secondary ICD10  Diagnoses – ICD10  Underlying (primary) cause of death: ICD10  Contributory (secondary) cause of death: ICD10 | I42.1, I42.2 | Obstructive hypertrophic cardiomyopathy, Other hypertrophic cardiomyopathy |
|  | 41203  41205  41271 | Diagnoses – main ICD9  Diagnoses – secondary ICD9  Diagnoses – ICD9 | 4251 | Hypertrophic obstructive cardiomyopathy |
|  | 41200  41210  41272 | Operative procedures – main OPCS4  Operative procedures – secondary OPCS4  Operative procedures - OPCS4 | K16.6, K24.5, K24.6, K24.7 | Percutaneous transluminal chemical mediated septal ablation, Relief of left ventricular outflow tract obstruction, Myectomy of LVOT, Myotomy of LVOT |
| Mitral valve disease | 20002 | Non-cancer illness code, self-reported | 1488, 1489 | Mitral valve prolapse, Mitral stenosis |
|  | 20004 | Operation code, self-reported | 1100 | Mitral valve repair/replacement |
|  | 41202  41204  41270  40001  40002 | Diagnoses – main ICD10  Diagnoses – secondary ICD10  Diagnoses – ICD10  Underlying (primary) cause of death: ICD10  Contributory (secondary) cause of death: ICD10 | I05.0, I05.1, I05.2, I05.8, I05.9, I34.0, I34.1, I34.2, I34.8, I34.9 | Mitral stenosis, Rheumatic mitral insufficiency, Mitral stenosis with insufficiency, Other mitral valve diseases, Mitral valve disease, unspecified, Mitral (valve) insufficiency, Mitral (valve) prolapse, Nonrheumatic mitral (valve) stenosis, Other nonrheumatic mitral valve disorders, Nonrheumatic mitral valve disorder, unspecified |
|  | 41203  41205  41271 | Diagnoses – main ICD9  Diagnoses – secondary ICD9  Diagnoses – ICD9 | 3940, 3942, 3949 | Mitral stenosis, Mitral stenosis with insufficiency, Other and unspecified diseases of mitral valve |
|  | 41200  41210  41272 | Operative procedures – main OPCS4  Operative procedures – secondary OPCS4  Operative procedures - OPCS4 | K25.1, K25.2, K25.3, K25.4, K25.5, K25.8, K25.9, K31.1, K32.1, K30.1 | Allograft replacement of mitral valve, Xenograft replacement of mitral valve, Prosthetic replacement of mitral valve, Replacement of mitral valve NEC23, Mitral valve repair NEC728, Other specified plastic repair of mitral valve, Unspecified plastic repair of mitral valve, Open mitral valvotomy, Closed mitral valvotomy, Revision of plastic repair of mitral valve |
|  | 41256  41258  41273 | Operative procedures – main OPCS3  Operative procedures – secondary OPCS3  Operative procedures – OPCS3 | 313.1, 314.1 | Heart valve replacement : mitral valve , Other valvuloplasty : mitral valve |
| Myocardial infarction | 20002 | Non-cancer illness code, self-reported | 1075 | Heart attack/myocardial infarction |
|  | 41202  41204  41270  40001  40002 | Diagnoses – main ICD10  Diagnoses – secondary ICD10  Diagnoses – ICD10  Underlying (primary) cause of death: ICD10  Contributory (secondary) cause of death: ICD10 | I21, I22, I23, I24.8, I24.9 | Acute myocardial infarction, Subsequent myocardial infarction, Certain current complications following acute myocardial infarction, Other forms of acute ischaemic heart disease, Acute ischaemic heart disease, unspecified |
|  | 41203  41205  41271 | Diagnoses – main ICD9  Diagnoses – secondary ICD9  Diagnoses – ICD9 | 410, 411, 412, 413, 414 | Acute myocardial infarction, Other acute and subacute forms of ischaemic heart disease |
| Narrow complex tachycardia | 20002 | Non-cancer illness code, self-reported | 1487 | SVT/ supraventricular tachycardia |
|  | 41202  41204  41270  40001  40002 | Diagnoses – main ICD10  Diagnoses – secondary ICD10  Diagnoses – ICD10  Underlying (primary) cause of death: ICD10  Contributory (secondary) cause of death: ICD10 | I47.1 | Supraventricular tachycardia |
|  | 41203  41205  41271 | Diagnoses – main ICD9  Diagnoses – secondary ICD9  Diagnoses – ICD9 | 4270 | Paroxysmal supraventricular tachycardia |
|  | 41200  41210  41272 | Operative procedures – main OPCS4  Operative procedures – secondary OPCS4  Operative procedures - OPCS4 | K57.2, K57.4, K57.5, K58.1, K58.2, K58.6 | Percutaneous transluminal ablation of conducting system of heart NEC, Percutaneous transluminal ablation of accessory pathway, Percutaneous transluminal ablation of atrial wall NEC, Percutaneous transluminal mapping of conducting system of heart NEC, Percutaneous transluminal electrophysiological studies on conducting system of heart, Percutaneous transluminal three dimensional electroanatomic mapping of conducting system of heart |
| Peripheral  vascular disease | 20002 | Non-cancer illness code, self-reported | 1087 | Leg claudication/ intermittent claudication |
|  | 20004 | Operation code, self-reported | 1102, 1103, 1104, 1108 | Fem-pop bypass/leg artery bypass, Leg artery aneurysm repair, Aortic aneurysm/repair or stent, Leg artery angioplasty +/- stent |
|  | 41202  41204  41270  40001  40002 | Diagnoses – main ICD10  Diagnoses – secondary ICD10  Diagnoses – ICD10  Underlying (primary) cause of death: ICD10  Contributory (secondary) cause of death: ICD10 | I70.0, I70.1, I70.2, I70.8, I70.9, I71.0, I71.1, I71.2, I71.3, I71.4, I71.5, I71.6, I71.8, I71.9 | Atherosclerosis of aorta, Atherosclerosis of renal artery, Atherosclerosis of arteries of the extremities, Atherosclerosis of other arteries, Generalised and unspecified atherosclerosis  Dissection of aorta [any part], Thoracic aortic aneurysm, rupture, Thoracic aortic aneurysm, without mention of rupture, Abdominal aortic aneurysm, ruptured, Abdominal aortic aneurysm, without mention of rupture, Thoracoabdominal aortic aneurysm, ruptured, Thoracoabdominal aortic aneurysm, without mention of rupture, Aortic aneurysm of unspecified site, ruptured, Aortic aneurysm of unspecified site, without mention of rupture |
|  | 41203  41205  41271 | Diagnoses – main ICD9  Diagnoses – secondary ICD9  Diagnoses – ICD9 | 4400, 4401, 4402, 4408, 4409, 4410, 4411, 4414, 4416 | Atherosclerosis of aorta, Atherosclerosis of renal artery, Atherosclerosis of arteries of the extremities, Atherosclerosis of other specified arteries, Atherosclerosis, generalized and unspecified, Dissecting aneurysm (any part), Thoracic aneurysm, ruptured, Abdominal aneurysm without mention of rupture, Aortic aneurysm of unspecified site without mention of rupture |
|  | 41200  41210  41272 | Operative procedures – main OPCS4  Operative procedures – secondary OPCS4  Operative procedures - OPCS4 | L16, L18, L19, L20, L21, L22, L23, L25, L26, L27, L28, L29, L30, L31, L37, L39, L48, L49, L50, L51, L52, L53, L54, L56, L57, L58, L59, L60, L62, L63 | Extra-anatomic bypass of aorta, Emergency replacement of aneurysmal segment of aorta, Other replacement of aneurysmal segment of aorta, Other emergency bypass of segment of aorta, Other bypass of segment of aorta, Attention to prosthesis of aorta, Plastic repair of aorta, Other open operations on aorta, Transluminal operations on aorta, Transluminal insertion of stent graft for aneurysmal segment of aorta, Transluminal operations on aneurysmal segment of aorta, Reconstruction of carotid artery, Other open operations on carotid artery, Transluminal operations on carotid artery, Reconstruction of subclavian artery, Transluminal operations on subclavian artery, Emergency replacement of aneurysmal iliac artery, Other replacement of aneurysmal iliac artery, Other emergency bypass of iliac artery, Other bypass of iliac artery, Reconstruction of iliac artery, Other open operations on iliac artery, Transluminal operations on iliac artery, Emergency replacement of aneurysmal femoral artery, Other replacement of aneurysmal femoral artery, Other emergency bypass of femoral artery, Other bypass of femoral artery, Reconstruction of femoral artery, Other open operations on femoral artery, Transluminal operations on femoral artery |
|  | 41256  41258  41273 | Operative procedures – main OPCS3  Operative procedures – secondary OPCS3  Operative procedures – OPCS3 | 881, 884 | Endarterectomy, Repair of artery |
| Ventricular arrhythmia | 41202  41204  41270  40001  40002 | Diagnoses – main ICD10  Diagnoses – secondary ICD10  Diagnoses – ICD10  Underlying (primary) cause of death: ICD10  Contributory (secondary) cause of death: ICD10 | I46.0, I46.1, I46.9, I47.0, I47.2, I49.0 | Cardiac arrest with successful resuscitation, Sudden cardiac death, so described, Cardiac arrest, unspecified, Reentry ventricular arrhythmia, Ventricular tachycardia, Ventricular fibrillation and flutter |
|  | 41203  41205  41271 | Diagnoses – main ICD9  Diagnoses – secondary ICD9  Diagnoses – ICD9 | 4271, 4274, 4275 | Paroxysmal ventricular tachycardia, Ventricular fibrillation and flutter, Cardiac arrest |
|  | 41200  41210  41272 | Operative procedures – main OPCS4  Operative procedures – secondary OPCS4  Operative procedures - OPCS4 | K57.6, K64.1, X50.3, X50.4, X50.8, X50.9 | Percutaneous transluminal ablation of ventricular wall, Percutaneous radiofrequency ablation of epicardium, External ventricular defibrillation, Other specified external resuscitation, Unspecified external resuscitation |

ICD: International Classification of Diseases; OPCS: Operating Procedure Codes Supplement.

# Table S2. Variance inflation factor of predictor variables in regression models 2 and 3 by endpoint and biomarker.

| **Outcome** | **Biomarker VIF** | | **Age** | **Sex** | **Ethnicity** | **Smoker** | **BMI** | **Hyperlipidemia** | **HTN** | **Diabetes** | **LVEDVi** | **LVEF** |
| --- | --- | --- | --- | --- | --- | --- | --- | --- | --- | --- | --- | --- |
| MACE | MadWT |  |  |  |  |  |  |  |  |  |  |  |
|  |  | 1.21 | 1.17 | 1.10 | 1.02 | 1.01 | 1.13 | 1.27 | 1.25 | 1.12 |  |  |
|  |  | 1.22 | 1.20 | 1.27 | 1.02 | 1.02 | 1.15 | 1.28 | 1.26 | 1.13 | 1.24 | 1.13 |
|  | MaxWT |  |  |  |  |  |  |  |  |  |  |  |
|  |  | 1.57 | 1.16 | 1.33 | 1.01 | 1.02 | 1.22 | 1.26 | 1.26 | 1.12 |  |  |
|  |  | 1.57 | 1.19 | 1.46 | 1.02 | 1.02 | 1.24 | 1.27 | 1.26 | 1.13 | 1.24 | 1.13 |
|  | MeanWT |  |  |  |  |  |  |  |  |  |  |  |
|  |  | 1.98 | 1.13 | 1.59 | 1.01 | 1.02 | 1.34 | 1.26 | 1.25 | 1.12 |  |  |
|  |  | 2.01 | 1.16 | 1.65 | 1.02 | 1.02 | 1.37 | 1.27 | 1.26 | 1.13 | 1.24 | 1.16 |
|  | LVMi |  |  |  |  |  |  |  |  |  |  |  |
|  |  | 1.34 | 1.13 | 1.33 | 1.02 | 1.01 | 1.12 | 1.27 | 1.25 | 1.12 |  |  |
|  |  | 2.53 | 1.15 | 1.28 | 1.02 | 1.02 | 1.18 | 1.27 | 1.24 | 1.13 | 2.23 | 1.21 |
| Heart failure | MadWT |  |  |  |  |  |  |  |  |  |  |  |
|  |  | 1.19 | 1.15 | 1.10 | 1.01 | 1.01 | 1.14 | 1.27 | 1.26 | 1.15 |  |  |
|  |  | 1.20 | 1.19 | 1.23 | 1.02 | 1.02 | 1.16 | 1.31 | 1.29 | 1.15 | 1.80 | 1.78 |
|  | MaxWT |  |  |  |  |  |  |  |  |  |  |  |
|  |  | 1.51 | 1.13 | 1.30 | 1.01 | 1.01 | 1.23 | 1.26 | 1.26 | 1.15 |  |  |
|  |  | 1.57 | 1.18 | 1.40 | 1.02 | 1.02 | 1.26 | 1.30 | 1.29 | 1.15 | 1.77 | 1.76 |
|  | MeanWT |  |  |  |  |  |  |  |  |  |  |  |
|  |  | 1.89 | 1.11 | 1.54 | 1.01 | 1.02 | 1.35 | 1.26 | 1.25 | 1.15 |  |  |
|  |  | 2.05 | 1.14 | 1.56 | 1.02 | 1.02 | 1.39 | 1.30 | 1.28 | 1.14 | 1.78 | 1.80 |
|  | LVMi |  |  |  |  |  |  |  |  |  |  |  |
|  |  | 1.27 | 1.11 | 1.25 | 1.01 | 1.01 | 1.13 | 1.26 | 1.24 | 1.15 |  |  |
|  |  | 3.42 | 1.13 | 1.26 | 1.02 | 1.02 | 1.17 | 1.29 | 1.26 | 1.14 | 3.34 | 1.80 |
| Arrhythmia | MadWT |  |  |  |  |  |  |  |  |  |  |  |
|  |  | 1.20 | 1.16 | 1.10 | 1.01 | 1.01 | 1.13 | 1.25 | 1.25 | 1.11 |  |  |
|  |  | 1.20 | 1.17 | 1.25 | 1.01 | 1.01 | 1.14 | 1.26 | 1.26 | 1.12 | 1.32 | 1.23 |
|  | MaxWT |  |  |  |  |  |  |  |  |  |  |  |
|  |  | 1.53 | 1.14 | 1.31 | 1.01 | 1.01 | 1.21 | 1.25 | 1.24 | 1.11 |  |  |
|  |  | 1.54 | 1.16 | 1.43 | 1.01 | 1.01 | 1.23 | 1.26 | 1.26 | 1.12 | 1.32 | 1.23 |
|  | MeanWT |  |  |  |  |  |  |  |  |  |  |  |
|  |  | 1.93 | 1.11 | 1.57 | 1.01 | 1.01 | 1.32 | 1.25 | 1.24 | 1.11 |  |  |
|  |  | 1.97 | 1.13 | 1.60 | 1.01 | 1.01 | 1.36 | 1.26 | 1.25 | 1.12 | 1.33 | 1.26 |
|  | LVMi |  |  |  |  |  |  |  |  |  |  |  |
|  |  | 1.35 | 1.11 | 1.34 | 1.01 | 1.01 | 1.11 | 1.25 | 1.24 | 1.11 |  |  |
|  |  | 2.79 | 1.12 | 1.30 | 1.01 | 1.01 | 1.17 | 1.26 | 1.25 | 1.12 | 2.48 | 1.28 |
| Death from any cause | MadWT |  |  |  |  |  |  |  |  |  |  |  |
|  |  | 1.20 | 1.15 | 1.10 | 1.01 | 1.02 | 1.13 | 1.25 | 1.23 | 1.11 |  |  |
|  |  | 1.21 | 1.18 | 1.29 | 1.01 | 1.02 | 1.14 | 1.25 | 1.24 | 1.12 | 1.24 | 1.12 |
|  | MaxWT |  |  |  |  |  |  |  |  |  |  |  |
|  |  | 1.59 | 1.14 | 1.35 | 1.01 | 1.02 | 1.22 | 1.25 | 1.24 | 1.11 |  |  |
|  |  | 1.60 | 1.17 | 1.52 | 1.01 | 1.02 | 1.24 | 1.25 | 1.24 | 1.12 | 1.23 | 1.12 |
|  | MeanWT |  |  |  |  |  |  |  |  |  |  |  |
|  |  | 2.09 | 1.11 | 1.69 | 1.01 | 1.02 | 1.34 | 1.25 | 1.23 | 1.12 |  |  |
|  |  | 2.12 | 1.14 | 1.81 | 1.01 | 1.02 | 1.37 | 1.25 | 1.24 | 1.12 | 1.24 | 1.13 |
|  | LVMi |  |  |  |  |  |  |  |  |  |  |  |
|  |  | 1.50 | 1.12 | 1.48 | 1.01 | 1.01 | 1.11 | 1.25 | 1.23 | 1.12 |  |  |
|  |  | 2.53 | 1.14 | 1.49 | 1.01 | 1.02 | 1.18 | 1.25 | 1.24 | 1.12 | 2.04 | 1.13 |
| Myocardial infarction | MadWT |  |  |  |  |  |  |  |  |  |  |  |
|  |  | 1.20 | 1.19 | 1.09 | 1.02 | 1.02 | 1.14 | 1.28 | 1.26 | 1.12 |  |  |
|  |  | 1.20 | 1.22 | 1.24 | 1.02 | 1.02 | 1.16 | 1.28 | 1.26 | 1.13 | 1.22 | 1.11 |
|  | MaxWT |  |  |  |  |  |  |  |  |  |  |  |
|  |  | 1.54 | 1.17 | 1.30 | 1.02 | 1.02 | 1.23 | 1.27 | 1.26 | 1.12 |  |  |
|  |  | 1.54 | 1.21 | 1.42 | 1.02 | 1.02 | 1.25 | 1.28 | 1.26 | 1.12 | 1.21 | 1.11 |
|  | MeanWT |  |  |  |  |  |  |  |  |  |  |  |
|  |  | 1.92 | 1.14 | 1.53 | 1.02 | 1.02 | 1.36 | 1.27 | 1.25 | 1.12 |  |  |
|  |  | 1.95 | 1.18 | 1.60 | 1.02 | 1.02 | 1.39 | 1.28 | 1.26 | 1.13 | 1.22 | 1.13 |
|  | LVMi |  |  |  |  |  |  |  |  |  |  |  |
|  |  | 1.35 | 1.15 | 1.33 | 1.02 | 1.02 | 1.12 | 1.28 | 1.25 | 1.12 |  |  |
|  |  | 2.35 | 1.16 | 1.30 | 1.02 | 1.02 | 1.20 | 1.28 | 1.25 | 1.12 | 2.06 | 1.15 |
| Stroke | MadWT |  |  |  |  |  |  |  |  |  |  |  |
|  |  | 1.23 | 1.16 | 1.11 | 1.01 | 1.01 | 1.13 | 1.26 | 1.25 | 1.13 |  |  |
|  |  | 1.23 | 1.19 | 1.30 | 1.02 | 1.01 | 1.14 | 1.26 | 1.25 | 1.13 | 1.27 | 1.15 |
|  | MaxWT |  |  |  |  |  |  |  |  |  |  |  |
|  |  | 1.59 | 1.15 | 1.35 | 1.01 | 1.01 | 1.21 | 1.25 | 1.25 | 1.13 |  |  |
|  |  | 1.60 | 1.18 | 1.49 | 1.02 | 1.01 | 1.23 | 1.26 | 1.25 | 1.13 | 1.27 | 1.16 |
|  | MeanWT |  |  |  |  |  |  |  |  |  |  |  |
|  |  | 2.02 | 1.12 | 1.64 | 1.01 | 1.01 | 1.32 | 1.25 | 1.24 | 1.13 |  |  |
|  |  | 2.06 | 1.15 | 1.69 | 1.02 | 1.01 | 1.35 | 1.26 | 1.25 | 1.13 | 1.28 | 1.18 |
|  | LVMi |  |  |  |  |  |  |  |  |  |  |  |
|  |  | 1.32 | 1.11 | 1.33 | 1.01 | 1.01 | 1.11 | 1.26 | 1.24 | 1.13 |  |  |
|  |  | 2.71 | 1.13 | 1.28 | 1.02 | 1.01 | 1.17 | 1.26 | 1.24 | 1.13 | 2.41 | 1.26 |
| CV death | MadWT |  |  |  |  |  |  |  |  |  |  |  |
|  |  | 1.17 | 1.13 | 1.09 | 1.01 | 1.02 | 1.13 | 1.23 | 1.23 | 1.12 |  |  |
|  |  | 1.19 | 1.15 | 1.20 | 1.02 | 1.03 | 1.14 | 1.25 | 1.24 | 1.13 | 1.33 | 1.29 |
|  | MaxWT |  |  |  |  |  |  |  |  |  |  |  |
|  |  | 1.47 | 1.12 | 1.27 | 1.01 | 1.02 | 1.21 | 1.23 | 1.23 | 1.12 |  |  |
|  |  | 1.50 | 1.14 | 1.33 | 1.02 | 1.03 | 1.22 | 1.25 | 1.24 | 1.13 | 1.34 | 1.31 |
|  | MeanWT |  |  |  |  |  |  |  |  |  |  |  |
|  |  | 1.79 | 1.10 | 1.46 | 1.01 | 1.02 | 1.32 | 1.23 | 1.21 | 1.12 |  |  |
|  |  | 1.87 | 1.12 | 1.42 | 1.02 | 1.03 | 1.35 | 1.25 | 1.23 | 1.12 | 1.39 | 1.39 |
|  | LVMi |  |  |  |  |  |  |  |  |  |  |  |
|  |  | 1.13 | 1.09 | 1.15 | 1.01 | 1.02 | 1.11 | 1.24 | 1.22 | 1.12 |  |  |
|  |  | 3.23 | 1.11 | 1.10 | 1.02 | 1.03 | 1.17 | 1.25 | 1.22 | 1.13 | 3.10 | 1.53 |

WT: wall thickness; LVMi: indexed left ventricular mass; CV: cardiovascular; CMR: cardiovascular magnetic resonance imaging; VIF: variance inflation factor; BMI: body mass index; HTN: hypertension; LVEDVi: indexed left ventricular end-diastolic volume; LVEF: left ventricular ejection fraction; MACE: major adverse cardiovascular endpoint; MadWT: mean absolute deviation of maximum segmental wall thickness; MaxWT: maximum end-diastolic wall thickness; MeanWT: mean end-diastolic wall thickness.

# Table S3. Multivariable regression models, demonstrating the relationship between CV risk factors, physical activity and *MadWT*.

|  | **Healthy + CV risk cohorts** | | | **Men** | | | **Women** | | |
| --- | --- | --- | --- | --- | --- | --- | --- | --- | --- |
|  | **ß** | **95% CI** | **P-value*** | **ß** | **95% CI** | **P-value*** | **ß** | **95% CI** | **P-value*** |
| **Age (per SD change)** | 0.074 | 0.071 - 0.077 | <0.001 | 0.062 | 0.057 – 0.067 | <0.001 | 0.081 | 0.078 – 0.085 | <0.001 |
| **Male** | 0.13 | 0.12 - 0.14 | <0.001 | - | - | - | - | - | - |
| **Non-White** | 0.059 | 0.043 – 0.075 | <0.001 | 0.042 | 0.017 – 0.068 | 0.004 | 0.070 | 0.050 – 0.091 | <0.001 |
| Asian | 0.041 | 0.019 – 0.064 | 0.001 | 0.029 | -0.0035 – 0.063 | 0.26 | 0.052 | 0.022 – 0.084 | 0.003 |
| Black | 0.11 | 0.077 – 0.14 | <0.001 | 0.075 | 0.026 – 0.13 | 0.010 | 0.13 | 0.090 – 0.17 | <0.001 |
| Mixed | 0.054 | -0.0072 – 0.12 | 0.30 | 0.071 | -0.050 – 0.21 | 0.86 | 0.052 | -0.018 – 0.13 | 0.50 |
| **Height (per SD change)** | 0.028 | 0.024 - 0.032 | <0.001 | 0.022 | 0.018 – 0.027 | <0.001 | 0.017 | 0.014 – 0.021 | <0.001 |
| **BMI (per SD change)** | 0.043 | 0.040 - 0.047 | <0.001 | 0.057 | 0.053 – 0.062 | <0.001 | 0.036 | 0.033 – 0.040 | <0.001 |
| **TPA (per SD change)** | 0.0061 | 0.0034 – 0.0088 | <0.001 | 0.0048 | 0.0004 – 0.0092 | 0.096 | 0.0053 | 0.0020 – 0.0087 | 0.005 |
| vigorous PA (per SD change) | 0.0093 | 0.0061 – 0.0124 | <0.001 | 0.013 | 0.0075 – 0.018 | <0.001 | 0.0052 | 0.0014 – 0.0092 | 0.020 |
| **Smoker** | 0.049 | 0.040 - 0.064 | <0.001 | 0.039 | 0.017 – 0.061 | 0.001 | 0.054 | 0.035 – 0.075 | <0.001 |
| **Hyperlipidemia** | 0.0041 | -0.0029 - 0.011 | 0.76 | 0.0015 | -0.0089 – 0.012 | 1.0 | 0.0056 | -0.0036 – 0.015 | 0.71 |
| **Hypertension** | 0.098 | 0.090 - 0.11 | <0.001 | 0.095 | 0.084 – 0.11 | <0.001 | 0.099 | 0.089 – 0.11 | <0.001 |
| **Diabetes** | 0.026 | 0.012 - 0.040 | 0.001 | 0.0061 | -0.013 – 0.026 | 1.0 | 0.045 | 0.025 – 0.066 | <0.001 |

CV: cardiovascular; MadWT: mean absolute deviation of maximum segmental wall thickness; SD: standard deviation; CI: confidence interval; BMI: body mass index; PA: physical activity; TPA: total physical activity in MET-min/week. *Multiplied by Bonferroni correction factor 3.

# Table S4. Results of Cox proportional hazards regression models to predict primary study endpoints by WT indices.

| **Outcome** | **Biomarker** | **Model** | **HR per SD** | **CI** | ***P-value** | **C-index before** | **C-index after** | **△C-index** | **C-index P-value** |
| --- | --- | --- | --- | --- | --- | --- | --- | --- | --- |
| MACE | MadWT | 1 | 1.54 | 1.45 - 1.63 | <0.001 | 0.500 | 0.634 | 0.134 | <0.001 |
|  |  | 2 | 1.28 | 1.19 - 1.38 | <0.001 | 0.692 | 0.700 | 0.008 | <0.001 |
|  |  | 3 | 1.28 | 1.19 - 1.37 | <0.001 | 0.696 | 0.702 | 0.007 | <0.001 |
|  |  | 4 | 1.13 | 1.04 - 1.23 | 0.005 | 0.707 | 0.709 | 0.001 | 0.003 |
|  | MaxWT | 1 | 1.66 | 1.55 - 1.77 | <0.001 | 0.500 | 0.649 | 0.149 | <0.001 |
|  |  | 2 | 1.38 | 1.26 - 1.51 | <0.001 | 0.692 | 0.702 | 0.010 | <0.001 |
|  |  | 3 | 1.37 | 1.25 - 1.49 | <0.001 | 0.696 | 0.703 | 0.008 | <0.001 |
|  |  | 4 | 1.11 | 0.98 - 1.25 | 0.11 | 0.707 | 0.708 | 0.001 | 0.003 |
|  | MeanWT | 1 | 1.69 | 1.58 - 1.80 | <0.001 | 0.500 | 0.651 | 0.152 | <0.001 |
|  |  | 2 | 1.54 | 1.40 - 1.70 | <0.001 | 0.692 | 0.702 | 0.010 | <0.001 |
|  |  | 3 | 1.50 | 1.36 - 1.65 | <0.001 | 0.696 | 0.704 | 0.009 | <0.001 |
|  |  | 4 | 1.20 | 1.00 - 1.44 | 0.045 | 0.707 | 0.707 | 0.000 | 0.99 |
|  | LVMi | 1 | 1.48 | 1.40 - 1.57 | <0.001 | 0.500 | 0.616 | 0.116 | <0.001 |
|  |  | 2 | 1.39 | 1.30 - 1.50 | <0.001 | 0.692 | 0.700 | 0.008 | <0.001 |
|  |  | 3 | 1.52 | 1.39 - 1.67 | <0.001 | 0.696 | 0.707 | 0.012 | <0.001 |
|  |  | 4 |  |  |  |  |  |  |  |
| Heart failure | MadWT | 1 | 1.64 | 1.49 - 1.80 | <0.001 | 0.500 | 0.670 | 0.170 | <0.001 |
|  |  | 2 | 1.30 | 1.16 - 1.46 | <0.001 | 0.760 | 0.769 | 0.009 | <0.001 |
|  |  | 3 | 1.30 | 1.15 - 1.47 | <0.001 | 0.824 | 0.827 | 0.003 | 0.007 |
|  |  | 4 | 1.15 | 1.01 - 1.32 | 0.039 | 0.826 | 0.827 | 0.001 | 0.92 |
|  | MaxWT | 1 | 1.88 | 1.69 - 2.09 | <0.001 | 0.500 | 0.696 | 0.196 | <0.001 |
|  |  | 2 | 1.51 | 1.31 - 1.74 | <0.001 | 0.760 | 0.773 | 0.014 | <0.001 |
|  |  | 3 | 1.42 | 1.23 - 1.63 | <0.001 | 0.824 | 0.827 | 0.003 | 0.003 |
|  |  | 4 | 1.18 | 0.96 - 1.44 | 0.11 | 0.826 | 0.827 | 0.001 | 0.51 |
|  | MeanWT | 1 | 1.95 | 1.76 - 2.16 | <0.001 | 0.500 | 0.688 | 0.188 | <0.001 |
|  |  | 2 | 1.79 | 1.55 - 2.08 | <0.001 | 0.760 | 0.775 | 0.015 | <0.001 |
|  |  | 3 | 1.52 | 1.30 - 1.77 | <0.001 | 0.824 | 0.826 | 0.003 | <0.001 |
|  |  | 4 | 1.23 | 0.92 - 1.65 | 0.15 | 0.826 | 0.827 | 0.000 | 0.41 |
|  | LVMi | 1 | 1.96 | 1.80 - 2.13 | <0.001 | 0.500 | 0.692 | 0.192 | <0.001 |
|  |  | 2 | 2.00 | 1.83 - 2.19 | <0.001 | 0.760 | 0.802 | 0.042 | <0.001 |
|  |  | 3 | 1.49 | 1.29 - 1.72 | <0.001 | 0.824 | 0.826 | 0.002 | 0.001 |
|  |  | 4 |  |  |  |  |  |  |  |
| Arrhythmia | MadWT | 1 | 1.60 | 1.52 - 1.68 | <0.001 | 0.500 | 0.639 | 0.139 | <0.001 |
|  |  | 2 | 1.33 | 1.25 - 1.41 | <0.001 | 0.700 | 0.711 | 0.011 | <0.001 |
|  |  | 3 | 1.33 | 1.26 - 1.42 | <0.001 | 0.715 | 0.723 | 0.009 | <0.001 |
|  |  | 4 | 1.26 | 1.18 - 1.35 | <0.001 | 0.720 | 0.725 | 0.005 | <0.001 |
|  | MaxWT | 1 | 1.74 | 1.65 - 1.84 | <0.001 | 0.500 | 0.665 | 0.165 | <0.001 |
|  |  | 2 | 1.48 | 1.38 - 1.60 | <0.001 | 0.700 | 0.716 | 0.016 | <0.001 |
|  |  | 3 | 1.46 | 1.36 - 1.57 | <0.001 | 0.715 | 0.727 | 0.012 | <0.001 |
|  |  | 4 | 1.42 | 1.29 - 1.57 | <0.001 | 0.720 | 0.727 | 0.007 | <0.001 |
|  | MeanWT | 1 | 1.74 | 1.65 - 1.84 | <0.001 | 0.500 | 0.659 | 0.159 | <0.001 |
|  |  | 2 | 1.65 | 1.53 - 1.79 | <0.001 | 0.700 | 0.718 | 0.018 | <0.001 |
|  |  | 3 | 1.56 | 1.44 - 1.69 | <0.001 | 0.715 | 0.729 | 0.014 | <0.001 |
|  |  | 4 | 1.88 | 1.61 - 2.19 | <0.001 | 0.720 | 0.730 | 0.011 | <0.001 |
|  | LVMi | 1 | 1.55 | 1.48 - 1.63 | <0.001 | 0.500 | 0.628 | 0.128 | <0.001 |
|  |  | 2 | 1.51 | 1.42 - 1.60 | <0.001 | 0.700 | 0.716 | 0.016 | <0.001 |
|  |  | 3 | 1.37 | 1.27 - 1.49 | <0.001 | 0.715 | 0.720 | 0.005 | <0.001 |
|  |  | 4 |  |  |  |  |  |  |  |
| Death from any cause | MadWT | 1 | 1.36 | 1.27 - 1.46 | <0.001 | 0.500 | 0.576 | 0.076 | <0.001 |
|  |  | 2 | 1.09 | 1.00 - 1.19 | 0.75 | 0.714 | 0.714 | 0.000 | 0.89 |
|  |  | 3 | 1.09 | 1.00 - 1.18 | 0.89 | 0.716 | 0.716 | 0.000 | 0.25 |
|  |  | 4 | 1.06 | 0.96 - 1.17 | 0.25 | 0.715 | 0.715 | 0.000 | 0.41 |
|  | MaxWT | 1 | 1.41 | 1.30 - 1.52 | <0.001 | 0.500 | 0.587 | 0.087 | <0.001 |
|  |  | 2 | 1.10 | 0.99 - 1.22 | 1.00 | 0.714 | 0.713 | 0.000 | 0.028 |
|  |  | 3 | 1.09 | 0.99 - 1.21 | 1.00 | 0.716 | 0.715 | -0.001 | 0.24 |
|  |  | 4 | 1.05 | 0.91 - 1.21 | 0.52 | 0.715 | 0.715 | 0.000 | 0.13 |
|  | MeanWT | 1 | 1.37 | 1.27 - 1.48 | <0.001 | 0.500 | 0.585 | 0.085 | <0.001 |
|  |  | 2 | 1.12 | 1.00 - 1.26 | 0.80 | 0.714 | 0.714 | 0.000 | 0.039 |
|  |  | 3 | 1.11 | 0.99 - 1.25 | 1.00 | 0.716 | 0.716 | 0.000 | 0.97 |
|  |  | 4 | 1.04 | 0.84 - 1.28 | 0.72 | 0.715 | 0.715 | 0.000 | 0.19 |
|  | LVMi | 1 | 1.20 | 1.12 - 1.30 | <0.001 | 0.500 | 0.540 | 0.040 | <0.001 |
|  |  | 2 | 1.09 | 0.99 - 1.20 | 1.00 | 0.714 | 0.713 | 0.000 | 0.50 |
|  |  | 3 | 1.13 | 0.99 - 1.28 | 1.00 | 0.716 | 0.715 | -0.001 | 0.22 |
|  |  | 4 |  |  |  |  |  |  |  |

WT: wall thickness; HR: hazard ratio; CI: confidence interval; MACE: major adverse cardiovascular endpoint; MadWT: mean absolute deviation of maximum segmental wall thickness; MaxWT: maximum end-diastolic wall thickness; MeanWT: mean end-diastolic wall thickness; LVMi: indexed left ventricular mass. *Models 1-3 multiplied by Bonferroni correction factor 16.

# Table S5. Results of Cox proportional hazards regression models to predict myocardial infarction, stroke and CV death by WT indices.

| **Outcome** | **Biomarker** | **Model** | **HR per SD** | **CI** | **P-value** | **C-index before** | **C-index after** | **△C-index** | **C-index P-value** |
| --- | --- | --- | --- | --- | --- | --- | --- | --- | --- |
| Myocardial infarction | MadWT | 1 | 1.53 | 1.40 - 1.66 | <0.001 | 0.500 | 0.634 | 0.134 | <0.001 |
|  |  | 2 | 1.23 | 1.11 - 1.36 | <0.001 | 0.710 | 0.713 | 0.003 | <0.001 |
|  |  | 3 | 1.23 | 1.11 - 1.36 | <0.001 | 0.713 | 0.716 | 0.003 | <0.001 |
|  |  | 4 | 1.08 | 0.96 - 1.21 | 0.22 | 0.723 | 0.723 | 0.000 | 0.099 |
|  | MaxWT | 1 | 1.72 | 1.57 - 1.88 | <0.001 | 0.500 | 0.669 | 0.169 | <0.001 |
|  |  | 2 | 1.34 | 1.18 - 1.52 | <0.001 | 0.710 | 0.716 | 0.007 | <0.001 |
|  |  | 3 | 1.33 | 1.18 - 1.51 | <0.001 | 0.713 | 0.719 | 0.005 | <0.001 |
|  |  | 4 | 1.07 | 0.90 - 1.28 | 0.44 | 0.723 | 0.723 | 0.000 | 0.73 |
|  | MeanWT | 1 | 1.79 | 1.64 - 1.96 | <0.001 | 0.500 | 0.677 | 0.177 | <0.001 |
|  |  | 2 | 1.50 | 1.31 - 1.72 | <0.001 | 0.710 | 0.719 | 0.009 | <0.001 |
|  |  | 3 | 1.47 | 1.29 - 1.69 | <0.001 | 0.713 | 0.721 | 0.008 | <0.001 |
|  |  | 4 | 1.20 | 0.93 - 1.54 | 0.17 | 0.723 | 0.723 | 0.000 | 0.79 |
|  | LVMi | 1 | 1.52 | 1.41 - 1.64 | <0.001 | 0.500 | 0.632 | 0.132 | <0.001 |
|  |  | 2 | 1.35 | 1.22 - 1.50 | <0.001 | 0.710 | 0.714 | 0.004 | <0.001 |
|  |  | 3 | 1.50 | 1.32 - 1.72 | <0.001 | 0.713 | 0.723 | 0.010 | <0.001 |
|  |  | 4 |  |  |  |  |  |  |  |
| Stroke | MadWT | 1 | 1.51 | 1.38 - 1.65 | <0.001 | 0.500 | 0.617 | 0.117 | <0.001 |
|  |  | 2 | 1.33 | 1.19 - 1.48 | <0.001 | 0.678 | 0.690 | 0.012 | <0.001 |
|  |  | 3 | 1.32 | 1.19 - 1.47 | <0.001 | 0.683 | 0.693 | 0.010 | <0.001 |
|  |  | 4 | 1.18 | 1.05 - 1.34 | 0.007 | 0.696 | 0.698 | 0.002 | 0.002 |
|  | MaxWT | 1 | 1.52 | 1.37 - 1.68 | <0.001 | 0.500 | 0.610 | 0.110 | <0.001 |
|  |  | 2 | 1.40 | 1.23 - 1.60 | <0.001 | 0.678 | 0.689 | 0.011 | <0.001 |
|  |  | 3 | 1.39 | 1.22 - 1.58 | <0.001 | 0.683 | 0.692 | 0.009 | <0.001 |
|  |  | 4 | 1.15 | 0.96 - 1.38 | 0.13 | 0.696 | 0.696 | 0.000 | 0.40 |
|  | MeanWT | 1 | 1.48 | 1.34 - 1.64 | <0.001 | 0.500 | 0.604 | 0.104 | <0.001 |
|  |  | 2 | 1.55 | 1.34 - 1.79 | <0.001 | 0.678 | 0.686 | 0.008 | <0.001 |
|  |  | 3 | 1.50 | 1.30 - 1.73 | <0.001 | 0.683 | 0.689 | 0.006 | <0.001 |
|  |  | 4 | 1.20 | 0.91 - 1.56 | 0.19 | 0.696 | 0.695 | -0.001 | 0.85 |
|  | LVMi | 1 | 1.37 | 1.25 - 1.50 | <0.001 | 0.500 | 0.580 | 0.080 | <0.001 |
|  |  | 2 | 1.39 | 1.26 - 1.54 | <0.001 | 0.678 | 0.689 | 0.011 | <0.001 |
|  |  | 3 | 1.52 | 1.32 - 1.74 | <0.001 | 0.683 | 0.696 | 0.013 | <0.001 |
|  |  | 4 |  |  |  |  |  |  |  |
| CV death | MadWT | 1 | 1.74 | 1.50 - 2.02 | <0.001 | 0.500 | 0.699 | 0.199 | <0.001 |
|  |  | 2 | 1.42 | 1.18 - 1.72 | <0.001 | 0.769 | 0.785 | 0.016 | <0.001 |
|  |  | 3 | 1.40 | 1.16 - 1.70 | 0.001 | 0.787 | 0.799 | 0.012 | <0.001 |
|  |  | 4 | 1.26 | 1.02 - 1.57 | 0.035 | 0.800 | 0.804 | 0.004 | 0.001 |
|  | MaxWT | 1 | 1.88 | 1.57 - 2.24 | <0.001 | 0.500 | 0.683 | 0.183 | <0.001 |
|  |  | 2 | 1.50 | 1.18 - 1.91 | 0.001 | 0.769 | 0.776 | 0.007 | <0.001 |
|  |  | 3 | 1.43 | 1.13 - 1.82 | 0.003 | 0.787 | 0.790 | 0.003 | 0.051 |
|  |  | 4 | 1.18 | 0.86 - 1.64 | 0.31 | 0.800 | 0.798 | -0.002 | 0.22 |
|  | MeanWT | 1 | 1.93 | 1.62 - 2.30 | <0.001 | 0.500 | 0.679 | 0.179 | <0.001 |
|  |  | 2 | 1.75 | 1.37 - 2.24 | <0.001 | 0.769 | 0.785 | 0.015 | <0.001 |
|  |  | 3 | 1.57 | 1.23 - 2.01 | <0.001 | 0.787 | 0.798 | 0.011 | <0.001 |
|  |  | 4 | 1.37 | 0.86 - 2.18 | 0.19 | 0.800 | 0.800 | 0.000 | 0.86 |
|  | LVMi | 1 | 1.62 | 1.42 - 1.84 | <0.001 | 0.500 | 0.652 | 0.152 | <0.001 |
|  |  | 2 | 1.57 | 1.35 - 1.82 | <0.001 | 0.769 | 0.781 | 0.011 | <0.001 |
|  |  | 3 | 1.49 | 1.18 - 1.87 | 0.001 | 0.787 | 0.800 | 0.013 | <0.001 |
|  |  | 4 |  |  |  |  |  |  |  |

WT: wall thickness; HR: hazard ratio; CI: confidence interval; MadWT: mean absolute deviation of maximum segmental wall thickness; MaxWT: maximum end-diastolic wall thickness; MeanWT: mean end-diastolic wall thickness; LVMi: indexed left ventricular mass.

# Table S6. Cox proportional hazards regression models to predict major study endpoints by WT indices in men and women.

|  | |  |  | **Men** | | | **Women** | | |
| --- | --- | --- | --- | --- | --- | --- | --- | --- | --- |
| **Outcome** | | **Biomarker** | **Model** | **HR per SD** | **CI** | **P-value** | **HR per SD** | **CI** | **P-value** |
|  | MACE | MadWT | 1 | 1.40 | 1.30 - 1.52 | <0.001 | 1.47 | 1.32 - 1.64 | <0.001 |
|  |  |  | 2 | 1.28 | 1.18 - 1.40 | <0.001 | 1.22 | 1.08 - 1.38 | 0.001 |
|  |  |  | 3 | 1.28 | 1.17 - 1.40 | <0.001 | 1.23 | 1.09 - 1.39 | 0.001 |
|  |  | MaxWT | 1 | 1.41 | 1.30 - 1.53 | <0.001 | 1.55 | 1.39 - 1.73 | <0.001 |
|  |  |  | 2 | 1.29 | 1.17 - 1.42 | <0.001 | 1.32 | 1.16 - 1.50 | <0.001 |
|  |  |  | 3 | 1.28 | 1.16 - 1.41 | <0.001 | 1.31 | 1.15 - 1.48 | <0.001 |
|  |  | MeanWT | 1 | 1.44 | 1.33 - 1.57 | <0.001 | 1.52 | 1.37 - 1.68 | <0.001 |
|  |  |  | 2 | 1.38 | 1.25 - 1.52 | <0.001 | 1.40 | 1.25 - 1.58 | <0.001 |
|  |  |  | 3 | 1.35 | 1.23 - 1.49 | <0.001 | 1.35 | 1.19 - 1.52 | <0.001 |
|  |  | LVMi | 1 | 1.30 | 1.20 - 1.41 | <0.001 | 1.28 | 1.19 - 1.38 | <0.001 |
|  |  |  | 2 | 1.33 | 1.23 - 1.45 | <0.001 | 1.25 | 1.16 - 1.35 | <0.001 |
|  |  |  | 3 | 1.49 | 1.34 - 1.65 | <0.001 | 1.24 | 1.09 - 1.40 | <0.001 |
|  | Heart failure | MadWT | 1 | 1.50 | 1.32 - 1.70 | <0.001 | 1.61 | 1.36 - 1.91 | <0.001 |
|  |  |  | 2 | 1.29 | 1.12 - 1.49 | <0.001 | 1.28 | 1.05 - 1.56 | 0.01 |
|  |  |  | 3 | 1.29 | 1.12 - 1.49 | 0.001 | 1.27 | 1.04 - 1.55 | 0.02 |
|  |  | MaxWT | 1 | 1.64 | 1.44 - 1.87 | <0.001 | 1.74 | 1.46 - 2.07 | <0.001 |
|  |  |  | 2 | 1.44 | 1.24 - 1.67 | <0.001 | 1.38 | 1.12 - 1.70 | 0.002 |
|  |  |  | 3 | 1.37 | 1.17 - 1.59 | <0.001 | 1.22 | 0.98 - 1.52 | 0.07 |
|  |  | MeanWT | 1 | 1.71 | 1.51 - 1.94 | <0.001 | 1.76 | 1.52 - 2.05 | <0.001 |
|  |  |  | 2 | 1.60 | 1.38 - 1.85 | <0.001 | 1.53 | 1.27 - 1.85 | <0.001 |
|  |  |  | 3 | 1.44 | 1.23 - 1.68 | <0.001 | 1.14 | 0.92 - 1.43 | 0.23 |
|  |  | LVMi | 1 | 1.75 | 1.57 - 1.95 | <0.001 | 1.77 | 1.60 - 1.95 | <0.001 |
|  |  |  | 2 | 1.80 | 1.62 - 2.01 | <0.001 | 1.71 | 1.55 - 1.89 | <0.001 |
|  |  |  | 3 | 1.40 | 1.18 - 1.65 | <0.001 | 1.22 | 1.00 - 1.49 | 0.05 |
|  | Arrhythmia | MadWT | 1 | 1.49 | 1.39 - 1.58 | <0.001 | 1.53 | 1.40 - 1.67 | <0.001 |
|  |  |  | 2 | 1.33 | 1.24 - 1.43 | <0.001 | 1.30 | 1.18 - 1.44 | <0.001 |
|  |  |  | 3 | 1.33 | 1.23 - 1.43 | <0.001 | 1.32 | 1.19 - 1.45 | <0.001 |
|  |  | MaxWT | 1 | 1.53 | 1.43 - 1.64 | <0.001 | 1.61 | 1.48 - 1.76 | <0.001 |
|  |  |  | 2 | 1.39 | 1.29 - 1.51 | <0.001 | 1.40 | 1.27 - 1.55 | <0.001 |
|  |  |  | 3 | 1.37 | 1.27 - 1.48 | <0.001 | 1.37 | 1.24 - 1.52 | <0.001 |
|  |  | MeanWT | 1 | 1.55 | 1.44 - 1.65 | <0.001 | 1.53 | 1.41 - 1.67 | <0.001 |
|  |  |  | 2 | 1.51 | 1.39 - 1.63 | <0.001 | 1.42 | 1.29 - 1.56 | <0.001 |
|  |  |  | 3 | 1.45 | 1.34 - 1.57 | <0.001 | 1.33 | 1.20 - 1.47 | <0.001 |
|  |  | LVMi | 1 | 1.37 | 1.28 - 1.47 | <0.001 | 1.37 | 1.29 - 1.46 | <0.001 |
|  |  |  | 2 | 1.43 | 1.34 - 1.53 | <0.001 | 1.34 | 1.26 - 1.43 | <0.001 |
|  |  |  | 3 | 1.35 | 1.23 - 1.49 | <0.001 | 1.18 | 1.06 - 1.31 | <0.001 |
|  | Death from any cause | MadWT | 1 | 1.26 | 1.14 - 1.38 | <0.001 | 1.31 | 1.16 - 1.47 | <0.001 |
|  |  |  | 2 | 1.07 | 0.96 - 1.19 | 0.22 | 1.11 | 0.98 - 1.27 | 0.11 |
|  |  |  | 3 | 1.07 | 0.96 - 1.19 | 0.23 | 1.11 | 0.97 - 1.27 | 0.12 |
|  |  | MaxWT | 1 | 1.24 | 1.12 - 1.37 | <0.001 | 1.28 | 1.14 - 1.45 | <0.001 |
|  |  |  | 2 | 1.07 | 0.95 - 1.19 | 0.26 | 1.11 | 0.96 - 1.27 | 0.16 |
|  |  |  | 3 | 1.06 | 0.95 - 1.19 | 0.30 | 1.11 | 0.96 - 1.28 | 0.15 |
|  |  | MeanWT | 1 | 1.20 | 1.08 - 1.32 | <0.001 | 1.20 | 1.06 - 1.35 | 0.004 |
|  |  |  | 2 | 1.08 | 0.96 - 1.22 | 0.19 | 1.10 | 0.95 - 1.27 | 0.19 |
|  |  |  | 3 | 1.07 | 0.95 - 1.20 | 0.27 | 1.10 | 0.95 - 1.27 | 0.20 |
|  |  | LVMi | 1 | 1.01 | 0.91 - 1.13 | 0.79 | 1.05 | 0.93 - 1.19 | 0.42 |
|  |  |  | 2 | 1.08 | 0.97 - 1.20 | 0.14 | 1.05 | 0.93 - 1.19 | 0.43 |
|  |  |  | 3 | 1.08 | 0.94 - 1.25 | 0.25 | 1.12 | 0.96 - 1.31 | 0.14 |

WT: wall thickness; HR: hazard ratio; CI: confidence interval; MACE: major adverse cardiovascular endpoint; MadWT: mean absolute deviation of maximum segmental wall thickness; MaxWT: maximum end-diastolic wall thickness; MeanWT: mean end-diastolic wall thickness; LVMi: indexed left ventricular mass.

# Table S7. WT & volumetric parameters in PSM hypertensive vs. non-hypertensive cohorts, stratified by total PA (MET-min/week).

| **Biomarker** | **Cohort** | **Cohort size (n)** | **Hypertension status** | **Median** | **95% CI** | **Absolute difference** | **Relative difference** | ***P-value** |
| --- | --- | --- | --- | --- | --- | --- | --- | --- |
| MadWT (mm) | All | 16,430 | No hypertension | 0.96 | 0.96 - 0.97 | 0.1 | 10.4 | <0.001 |
|  |  |  | Hypertension | 1.06 | 1.05 - 1.07 |  |  |  |
|  | Top 10% | 1,660 | No hypertension | 0.95 | 0.94 - 0.98 | 0.1 | 12.1 | <0.001 |
|  |  |  | Hypertension | 1.07 | 1.05 - 1.10 |  |  |  |
|  | Top 1% | 300 | No hypertension | 0.96 | 0.89 - 1.00 | 0.1 | 13.5 | 0.013 |
|  |  |  | Hypertension | 1.09 | 1.01 - 1.13 |  |  |  |
| MaxWT (mm) | All | 16,430 | No hypertension | 9.44 | 9.41 - 9.49 | 0.5 | 5.4 | <0.001 |
|  |  |  | Hypertension | 9.95 | 9.90 - 9.99 |  |  |  |
|  | Top 10% | 1,660 | No hypertension | 9.45 | 9.37 - 9.57 | 0.5 | 4.8 | <0.001 |
|  |  |  | Hypertension | 9.90 | 9.82 - 10.0 |  |  |  |
|  | Top 1% | 300 | No hypertension | 9.39 | 9.08 - 9.71 | 0.5 | 5.7 | 0.082 |
|  |  |  | Hypertension | 9.92 | 9.59 - 10.2 |  |  |  |
| MeanWT (mm) | All | 16,430 | No hypertension | 7.20 | 7.17 - 7.23 | 0.3 | 4.4 | <0.001 |
|  |  |  | Hypertension | 7.51 | 7.48 - 7.54 |  |  |  |
|  | Top 10% | 1,660 | No hypertension | 7.22 | 7.12 - 7.30 | 0.2 | 3.2 | <0.001 |
|  |  |  | Hypertension | 7.45 | 7.37 - 7.57 |  |  |  |
|  | Top 1% | 300 | No hypertension | 7.13 | 6.90 - 7.37 | 0.4 | 5.1 | 0.083 |
|  |  |  | Hypertension | 7.50 | 7.26 - 7.74 |  |  |  |
| LVMi (g/m^2) | All | 16,430 | No hypertension | 44.6 | 44.4 - 44.9 | 2.4 | 5.3 | <0.001 |
|  |  |  | Hypertension | 47.0 | 46.7 - 47.2 |  |  |  |
|  | Top 10% | 1,660 | No hypertension | 45.5 | 44.9 - 46.3 | 1.9 | 3.9 | <0.001 |
|  |  |  | Hypertension | 47.3 | 46.6 - 48.1 |  |  |  |
|  | Top 1% | 300 | No hypertension | 46.5 | 44.8 - 48.9 | 2.0 | 4.3 | 0.29 |
|  |  |  | Hypertension | 48.5 | 45.8 - 50.7 |  |  |  |
| LVEDVi (mL/m^2) | All | 16,430 | No hypertension | 75.6 | 75.2 - 76.0 | 1.0 | 1.4 | <0.001 |
|  |  |  | Hypertension | 76.6 | 76.2 - 77.1 |  |  |  |
|  | Top 10% | 1,660 | No hypertension | 77.5 | 76.5 - 78.7 | 0.3 | 0.4 | 1.0 |
|  |  |  | Hypertension | 77.9 | 76.5 - 78.9 |  |  |  |
|  | Top 1% | 300 | No hypertension | 78.1 | 74.3 - 81.5 | 0.4 | 0.5 | 1.0 |
|  |  |  | Hypertension | 78.5 | 75.8 - 80.6 |  |  |  |
| LVMVR (g/mL) | All | 16,430 | No hypertension | 0.59 | 0.59 - 0.59 | 0.02 | 3.7 | <0.001 |
|  |  |  | Hypertension | 0.61 | 0.61 - 0.61 |  |  |  |
|  | Top 10% | 1,660 | No hypertension | 0.58 | 0.57 - 0.59 | 0.03 | 4.8 | <0.001 |
|  |  |  | Hypertension | 0.61 | 0.60 - 0.61 |  |  |  |
|  | Top 1% | 300 | No hypertension | 0.58 | 0.56 - 0.59 | 0.03 | 4.9 | 0.042 |
|  |  |  | Hypertension | 0.61 | 0.60 - 0.63 |  |  |  |
| LVEF (%) | All | 16,430 | No hypertension | 59.5 | 59.3 - 59.7 | 0.8 | 1.4 | <0.001 |
|  |  |  | Hypertension | 60.4 | 60.2 - 60.5 |  |  |  |
|  | Top 10% | 1,660 | No hypertension | 59.0 | 58.6 - 59.6 | 1.5 | 2.5 | 0.053 |
|  |  |  | Hypertension | 60.5 | 59.8 - 61.0 |  |  |  |
|  | Top 1% | 300 | No hypertension | 59.3 | 58.6 - 60.8 | 1.3 | 2.1 | 1.0 |
|  |  |  | Hypertension | 60.7 | 58.3 - 61.7 |  |  |  |
| GLS (%) | All | 16,321 | No hypertension | 17.9 | 17.9 - 18.0 | 0.0 | 0.1 | 1.0 |
|  |  |  | Hypertension | 17.9 | 17.9 - 18.0 |  |  |  |
|  | Top 10% | 1,651 | No hypertension | 18.0 | 17.9 - 18.1 | 0.3 | 1.5 | 1.0 |
|  |  |  | Hypertension | 17.7 | 17.6 - 17.9 |  |  |  |
|  | Top 1% | 299 | No hypertension | 17.8 | 17.1 - 18.2 | 0.2 | 1.1 | 1.0 |
|  |  |  | Hypertension | 17.6 | 17.3 - 18.4 |  |  |  |
| Native T1 (ms) | All | 15,516 | No hypertension | 929 | 928 - 930 | 1.5 | 0.2 | 0.023 |
|  |  |  | Hypertension | 927 | 927 - 928 |  |  |  |
|  | Top 10% | 1,581 | No hypertension | 926 | 923 - 930 | 0.6 | 0.1 | 1.0 |
|  |  |  | Hypertension | 927 | 924 - 930 |  |  |  |
|  | Top 1% | 285 | No hypertension | 925 | 919 - 935 | 5.5 | 0.6 | 1.0 |
|  |  |  | Hypertension | 919 | 912 - 929 |  |  |  |

WT: wall thickness; PSM: propensity score matching; PA: physical activity; MET: metabolic equivalent of task; CI: confidence interval; MadWT: mean absolute deviation of maximum segmental wall thickness; MaxWT: maximum end-diastolic wall thickness; MeanWT: mean end-diastolic wall thickness; LVMi: indexed left ventricular mass; LVEDVi: indexed left ventricular end-diastolic volume; LVMVR: left ventricular mass to volume ratio; LVEF: left ventricular ejection fraction; GLS: global longitudinal strain. *Multiplied by Bonferroni correction factor 9.

# Table S8. WT & volumetric parameters in PSM hypertensive vs. non-hypertensive cohorts, stratified by vigorous PA (MET-min/week).

| **Biomarker** | **Cohort** | **Cohort size (n)** | **Hypertension status** | **Median** | **95% CI** | **Absolute difference** | **Relative difference** | ***P-value** |
| --- | --- | --- | --- | --- | --- | --- | --- | --- |
| MadWT (mm) | All | 12,282 | No hypertension | 0.97 | 0.96 - 0.97 | 0.1 | 10.8 | <0.001 |
|  |  |  | Hypertension | 1.07 | 1.06 - 1.08 |  |  |  |
|  | Top 10% | 1,130 | No hypertension | 1.00 | 0.97 - 1.03 | 0.1 | 9.6 | <0.001 |
|  |  |  | Hypertension | 1.10 | 1.06 - 1.12 |  |  |  |
|  | Top 1% | 236 | No hypertension | 1.01 | 0.96 - 1.06 | 0.1 | 10.2 | 0.023 |
|  |  |  | Hypertension | 1.11 | 1.08 - 1.18 |  |  |  |
| MaxWT (mm) | All | 12,282 | No hypertension | 9.48 | 9.44 - 9.52 | 0.5 | 5.3 | <0.001 |
|  |  |  | Hypertension | 9.99 | 9.94 - 10.0 |  |  |  |
|  | Top 10% | 1,130 | No hypertension | 9.88 | 9.61 - 10.0 | 0.3 | 2.9 | <0.001 |
|  |  |  | Hypertension | 10.2 | 10.0 - 10.3 |  |  |  |
|  | Top 1% | 236 | No hypertension | 9.75 | 9.44 - 10.2 | 0.7 | 6.7 | 0.048 |
|  |  |  | Hypertension | 10.4 | 9.85 - 10.9 |  |  |  |
| MeanWT (mm) | All | 12,282 | No hypertension | 7.23 | 7.19 - 7.25 | 0.3 | 4.5 | <0.001 |
|  |  |  | Hypertension | 7.55 | 7.52 - 7.58 |  |  |  |
|  | Top 10% | 1,130 | No hypertension | 7.44 | 7.33 - 7.56 | 0.3 | 3.5 | <0.001 |
|  |  |  | Hypertension | 7.70 | 7.59 - 7.83 |  |  |  |
|  | Top 1% | 236 | No hypertension | 7.42 | 7.22 - 7.71 | 0.3 | 4.5 | 0.21 |
|  |  |  | Hypertension | 7.76 | 7.43 - 7.98 |  |  |  |
| LVMi (g/m^2) | All | 12,282 | No hypertension | 45.1 | 44.9 - 45.4 | 2.6 | 5.7 | <0.001 |
|  |  |  | Hypertension | 47.7 | 47.4 - 47.9 |  |  |  |
|  | Top 10% | 1,130 | No hypertension | 48.7 | 47.7 - 49.6 | 1.9 | 3.9 | 0.010 |
|  |  |  | Hypertension | 50.6 | 49.3 - 51.6 |  |  |  |
|  | Top 1% | 236 | No hypertension | 49.4 | 47.6 - 50.8 | 2.1 | 4.3 | 0.84 |
|  |  |  | Hypertension | 51.5 | 49.2 - 53.5 |  |  |  |
| LVEDVi (mL/m^2) | All | 12,282 | No hypertension | 76.8 | 76.5 - 77.2 | 0.9 | 1.2 | 0.002 |
|  |  |  | Hypertension | 77.7 | 77.4 - 78.2 |  |  |  |
|  | Top 10% | 1,130 | No hypertension | 81.5 | 80.1 - 83.3 | 0.8 | 1.0 | 1.0 |
|  |  |  | Hypertension | 82.3 | 80.9 - 83.7 |  |  |  |
|  | Top 1% | 236 | No hypertension | 81.5 | 77.2 - 85.7 | 1.7 | 2.1 | 1.0 |
|  |  |  | Hypertension | 83.2 | 80.2 - 85.1 |  |  |  |
| LVMVR (g/mL) | All | 12,282 | No hypertension | 0.59 | 0.58 - 0.59 | 0.02 | 4.1 | <0.001 |
|  |  |  | Hypertension | 0.61 | 0.61 - 0.61 |  |  |  |
|  | Top 10% | 1,130 | No hypertension | 0.59 | 0.58 - 0.6 | 0.02 | 3.7 | <0.001 |
|  |  |  | Hypertension | 0.61 | 0.6 - 0.62 |  |  |  |
|  | Top 1% | 236 | No hypertension | 0.59 | 0.57 - 0.6 | 0.02 | 2.8 | 1.0 |
|  |  |  | Hypertension | 0.6 | 0.58 - 0.62 |  |  |  |
| LVEF (%) | All | 12,282 | No hypertension | 59.6 | 59.4 - 59.8 | 0.6 | 1.0 | <0.001 |
|  |  |  | Hypertension | 60.2 | 60.0 - 60.4 |  |  |  |
|  | Top 10% | 1,130 | No hypertension | 59.3 | 58.4 - 59.8 | 0.9 | 1.6 | 0.29 |
|  |  |  | Hypertension | 60.2 | 59.3 - 61.0 |  |  |  |
|  | Top 1% | 236 | No hypertension | 58.1 | 56.8 - 59.3 | 0.7 | 1.1 | 1.0 |
|  |  |  | Hypertension | 58.8 | 57.0 - 61.0 |  |  |  |
| GLS (%) | All | 12,282 | No hypertension | 18.0 | 17.9 - 18.0 | 0.1 | 0.3 | 1.0 |
|  |  |  | Hypertension | 17.9 | 17.8 - 18.0 |  |  |  |
|  | Top 10% | 1,130 | No hypertension | 17.9 | 17.7 - 18.1 | 0.0 | 0.2 | 1.0 |
|  |  |  | Hypertension | 17.9 | 17.7 - 18.2 |  |  |  |
|  | Top 1% | 236 | No hypertension | 18.0 | 17.6 - 18.4 | 0.2 | 1.1 | 1.0 |
|  |  |  | Hypertension | 17.8 | 17.2 - 18.6 |  |  |  |
| Native T1 (ms) | All | 12,282 | No hypertension | 927 | 926 - 928 | 1.2 | 0.1 | 0.69 |
|  |  |  | Hypertension | 926 | 925 - 927 |  |  |  |
|  | Top 10% | 1,130 | No hypertension | 921 | 919 - 924 | 1.4 | 0.2 | 1.0 |
|  |  |  | Hypertension | 920 | 917 - 923 |  |  |  |
|  | Top 1% | 236 | No hypertension | 918 | 914 - 926 | 1.5 | 0.2 | 1.0 |
|  |  |  | Hypertension | 920 | 915 - 928 |  |  |  |

WT: wall thickness; PSM: propensity score matching; PA: physical activity; MET: metabolic equivalent of task; CI: confidence interval; MadWT: mean absolute deviation of maximum segmental wall thickness; MaxWT: maximum end-diastolic wall thickness; MeanWT: mean end-diastolic wall thickness; LVMi: indexed left ventricular mass; LVEDVi: indexed left ventricular end-diastolic volume; LVMVR: left ventricular mass to volume ratio; LVEF: left ventricular ejection fraction; GLS: global longitudinal strain. *Multiplied by Bonferroni correction factor 9.

# Table S9: WT & volumetric parameters in PSM HTN vs. non-HTN cohorts, stratified by mean acceleration vector (milli-gravity).

| **Biomarker** | **Cohort** | **Cohort size (n)** | **Hypertension status** | **Median** | **95% CI** | **Absolute difference** | **Relative difference** | ***P-value** |
| --- | --- | --- | --- | --- | --- | --- | --- | --- |
| MadWT (mm) | All | 6,794 | No hypertension | 0.97 | 0.96 - 0.98 | 0.1 | 8.6 | <0.001 |
|  |  |  | Hypertension | 1.06 | 1.04 - 1.07 |  |  |  |
|  | Top 10% | 406 | No hypertension | 0.97 | 0.91 - 1.02 | 0.1 | 8.9 | 0.006 |
|  |  |  | Hypertension | 1.06 | 1.01 - 1.09 |  |  |  |
|  | Top 1% | 62 | No hypertension | 0.93 | 0.86 - 1.03 | 0.2 | 21.2 | 0.009 |
|  |  |  | Hypertension | 1.13 | 1.04 - 1.29 |  |  |  |
| MaxWT (mm) | All | 6,794 | No hypertension | 9.37 | 9.32 - 9.43 | 0.5 | 5.4 | <0.001 |
|  |  |  | Hypertension | 9.88 | 9.81 - 9.95 |  |  |  |
|  | Top 10% | 406 | No hypertension | 9.32 | 9.03 - 9.64 | 0.5 | 5.0 | 0.002 |
|  |  |  | Hypertension | 9.78 | 9.42 - 10.1 |  |  |  |
|  | Top 1% | 62 | No hypertension | 9.29 | 8.59 - 10.0 | 1.0 | 11.0 | 0.17 |
|  |  |  | Hypertension | 10.3 | 9.23 - 11.2 |  |  |  |
| MeanWT (mm) | All | 6,794 | No hypertension | 7.12 | 7.08 - 7.17 | 0.3 | 4.6 | <0.001 |
|  |  |  | Hypertension | 7.45 | 7.39 - 7.50 |  |  |  |
|  | Top 10% | 406 | No hypertension | 7.08 | 6.86 - 7.26 | 0.1 | 1.8 | 0.013 |
|  |  |  | Hypertension | 7.21 | 7.06 - 7.55 |  |  |  |
|  | Top 1% | 62 | No hypertension | 7.11 | 6.58 - 7.44 | 0.6 | 8.1 | 0.20 |
|  |  |  | Hypertension | 7.69 | 6.95 - 8.48 |  |  |  |
| LVMi (g/m^2) | All | 6,794 | No hypertension | 44.4 | 44.1 - 44.7 | 2.4 | 5.4 | <0.001 |
|  |  |  | Hypertension | 46.8 | 46.4 - 47.2 |  |  |  |
|  | Top 10% | 406 | No hypertension | 46.7 | 45.7 - 48.2 | 1.8 | 3.8 | 0.050 |
|  |  |  | Hypertension | 48.5 | 46.9 - 50.7 |  |  |  |
|  | Top 1% | 62 | No hypertension | 47.0 | 44.0 - 55.7 | 7.5 | 16.0 | 0.32 |
|  |  |  | Hypertension | 54.5 | 48.3 - 62.8 |  |  |  |
| LVEDVi (mL/m^2) | All | 6,794 | No hypertension | 76.1 | 75.5 - 76.7 | 0.5 | 0.7 | 0.36 |
|  |  |  | Hypertension | 76.6 | 76.1 - 77.3 |  |  |  |
|  | Top 10% | 406 | No hypertension | 82.6 | 79.9 - 85.3 | 1.3 | 1.6 | 1.0 |
|  |  |  | Hypertension | 83.9 | 82.1 - 87.6 |  |  |  |
|  | Top 1% | 62 | No hypertension | 86.0 | 81.3 - 92.8 | 5.9 | 6.9 | 1.0 |
|  |  |  | Hypertension | 91.9 | 82.6 - 101 |  |  |  |
| LVMVR (g/mL) | All | 6,794 | No hypertension | 0.58 | 0.58 - 0.59 | 0.0 | 4.7 | <0.001 |
|  |  |  | Hypertension | 0.61 | 0.61 - 0.61 |  |  |  |
|  | Top 10% | 406 | No hypertension | 0.57 | 0.55 - 0.58 | 0.0 | 3.8 | 0.015 |
|  |  |  | Hypertension | 0.59 | 0.58 - 0.60 |  |  |  |
|  | Top 1% | 62 | No hypertension | 0.55 | 0.54 - 0.57 | 0.0 | 6.5 | 1.0 |
|  |  |  | Hypertension | 0.59 | 0.53 - 0.65 |  |  |  |
| LVEF (%) | All | 6,794 | No hypertension | 59.6 | 59.3 - 59.8 | 0.9 | 1.5 | <0.001 |
|  |  |  | Hypertension | 60.5 | 60.2 - 60.8 |  |  |  |
|  | Top 10% | 406 | No hypertension | 59.0 | 57.6 - 59.7 | 0.3 | 0.5 | 1.0 |
|  |  |  | Hypertension | 59.3 | 58.3 - 60.3 |  |  |  |
|  | Top 1% | 62 | No hypertension | 59.2 | 55.9 - 62.3 | 0.9 | 1.5 | 1.0 |
|  |  |  | Hypertension | 60.0 | 55.9 - 63.1 |  |  |  |
| GLS (%) | All | 6,759 | No hypertension | 18.0 | 17.9 - 18.0 | 0.1 | 0.6 | 1.0 |
|  |  |  | Hypertension | 18.1 | 18.0 - 18.2 |  |  |  |
|  | Top 10% | 405 | No hypertension | 18.3 | 18.0 - 18.8 | 0.5 | 2.9 | 1.0 |
|  |  |  | Hypertension | 17.8 | 17.5 - 18.4 |  |  |  |
|  | Top 1% | 62 | No hypertension | 18.6 | 18.0 - 19.7 | 1.1 | 6.0 | 1.0 |
|  |  |  | Hypertension | 17.5 | 16.9 - 18.8 |  |  |  |
| Native T1 (ms) | All | 6,444 | No hypertension | 929 | 928 - 931 | 2.7 | 0.3 | 0.010 |
|  |  |  | Hypertension | 927 | 925 - 928 |  |  |  |
|  | Top 10% | 388 | No hypertension | 928 | 921 - 933 | 5.2 | 0.6 | 1.0 |
|  |  |  | Hypertension | 923 | 920 - 927 |  |  |  |
|  | Top 1% | 59 | No hypertension | 930 | 916 - 940 | 15.7 | 1.7 | 0.55 |
|  |  |  | Hypertension | 915 | 906 - 924 |  |  |  |

WT: wall thickness; PSM: propensity score matching; HTN: hypertension; PA: physical activity; CI: confidence interval; MadWT: mean absolute deviation of maximum segmental wall thickness; MaxWT: maximum end-diastolic wall thickness; MeanWT: mean end-diastolic wall thickness; LVMi: indexed left ventricular mass; LVEDVi: indexed left ventricular end-diastolic volume; LVMVR: left ventricular mass to volume ratio; LVEF: left ventricular ejection fraction; GLS: global longitudinal strain. *Multiplied by Bonferroni correction factor 9.

**Figures**


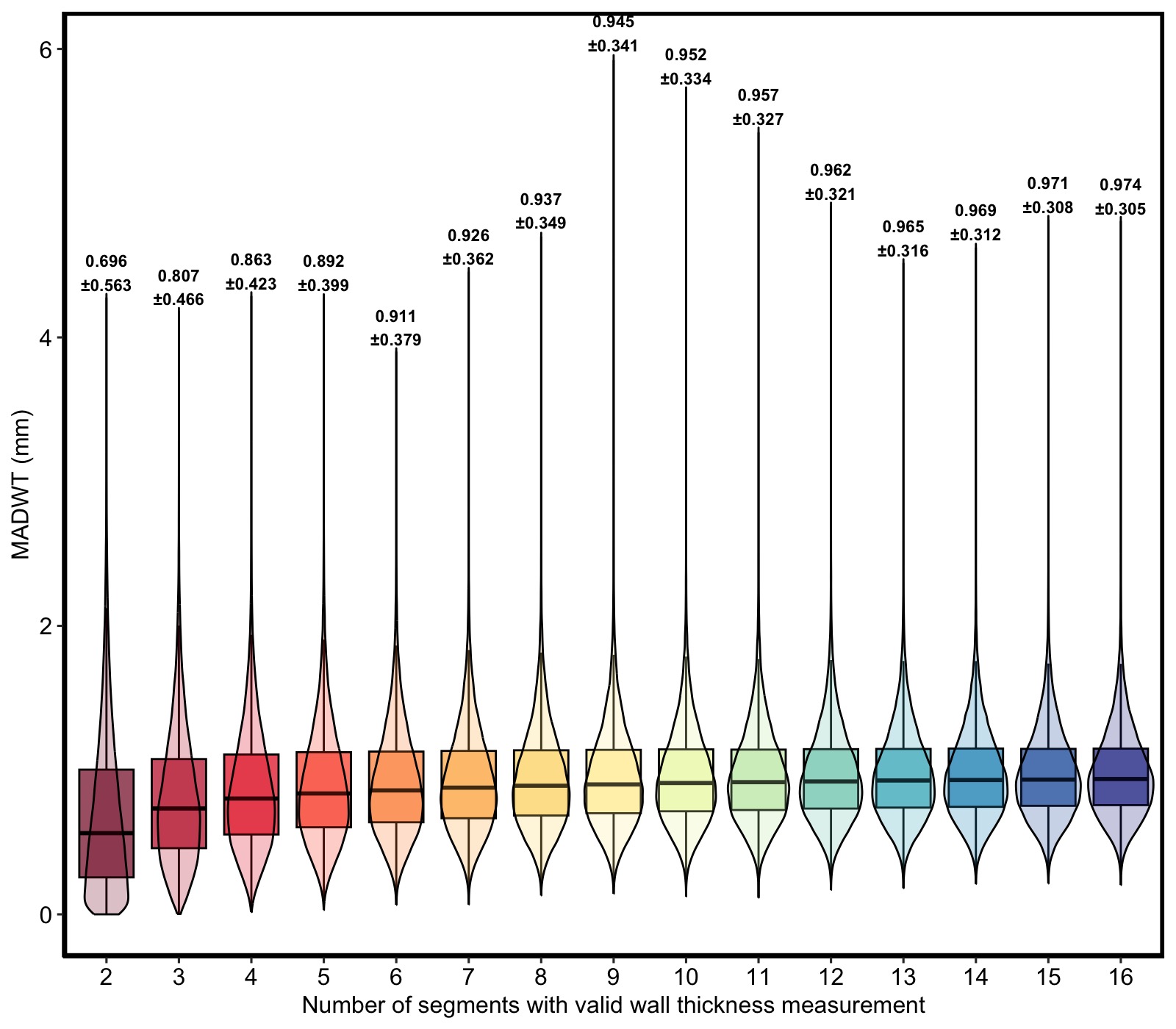


# Figure S1. *MadWT* distribution, stratified by number of measured LV segments.

The central tendency and spread of MadWT started to deviate and increase with ≤11 segments with valid wall thickness measurements.

Data presented as mean ± standard deviation (SD) and a combined violin-box plot. MadWT: mean absolute deviation of maximum segmental wall thickness; LV: left ventricle.

#
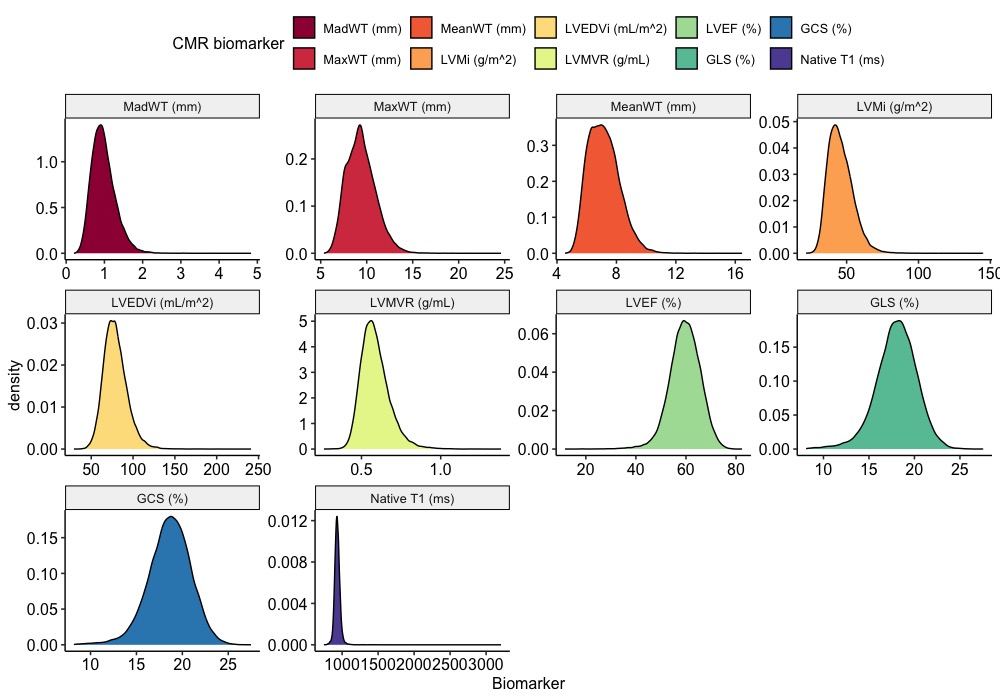

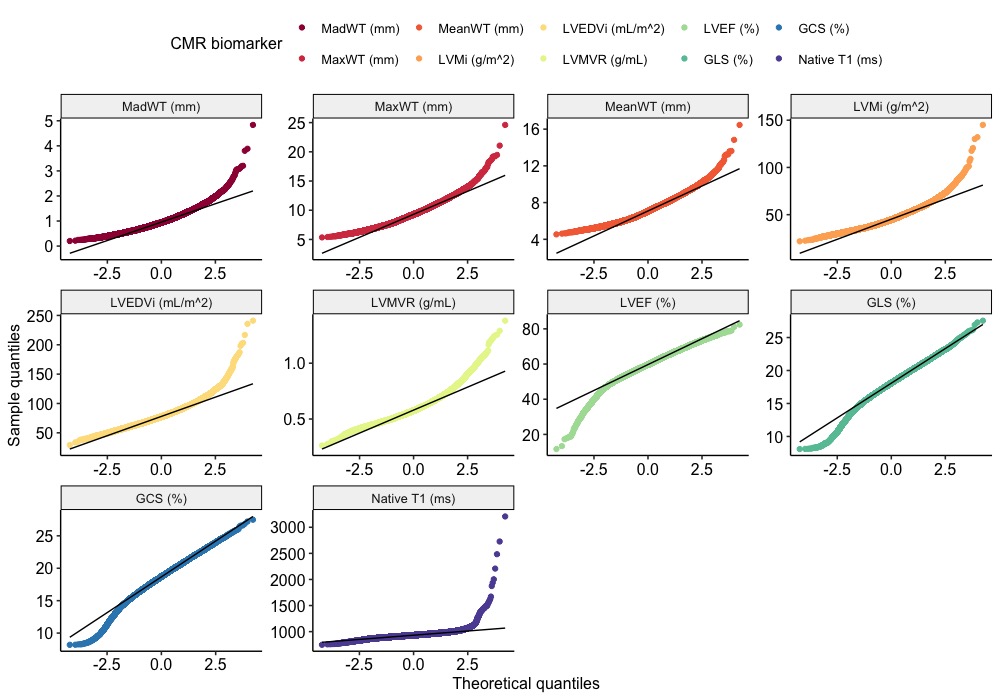
Figure S2. Density (A) and QQ (B) plots of CMR-derived biomarkers.

**B)**

**A)**

Excluding GLS and GCS, CMR-derived biomarkers from participants of the UK Biobank had a right-skewed distribution.


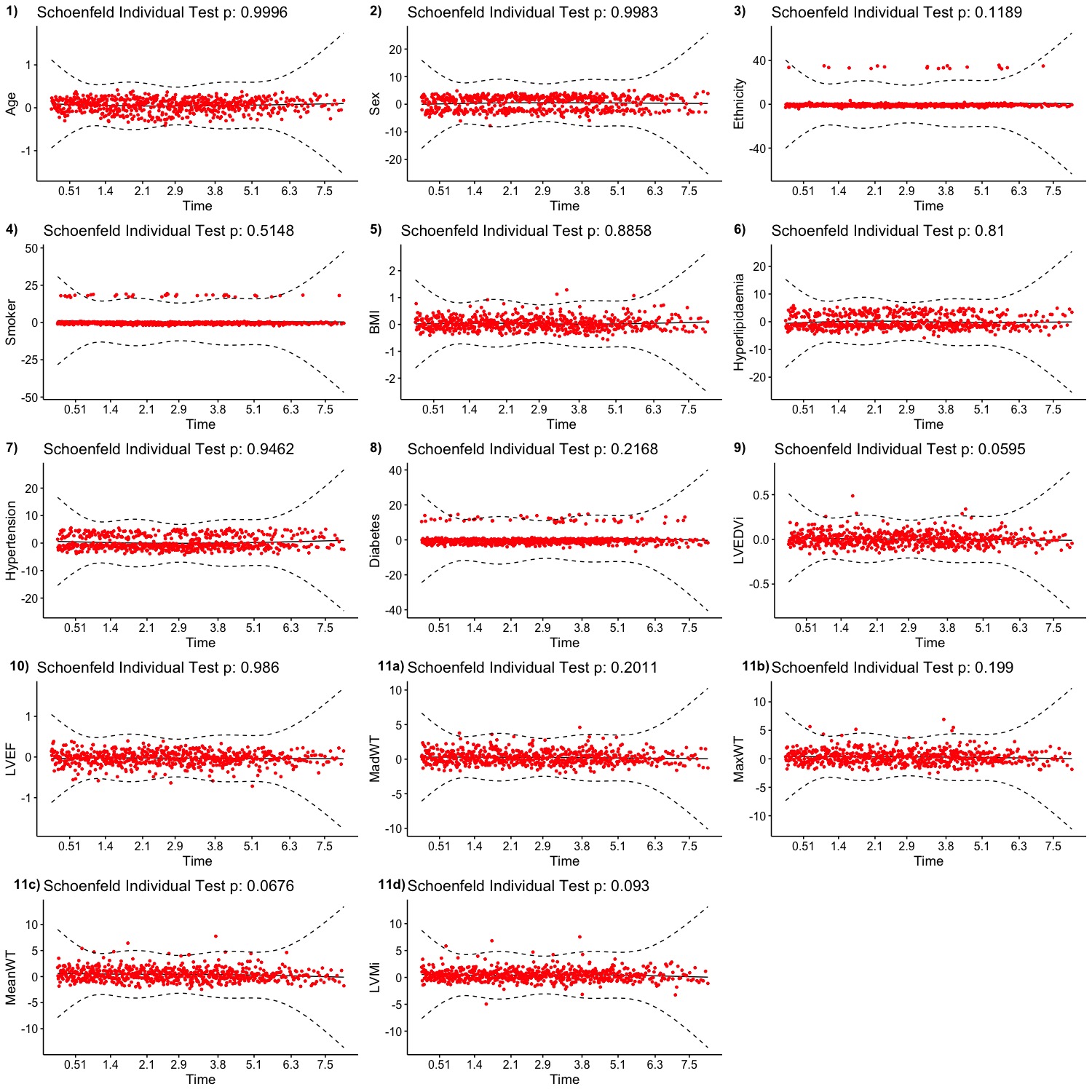


# Figure S3. Incident MACE covariate residual *vs.* time plots.

In the fully adjusted incident MACE Cox regression model, assumption of proportional hazards is supported for *MadWT, MaxWT, MeanWT, LVMi* and their covariates graphically.

LVEDVi: indexed left ventricular end-diastolic volume; LVEF: left ventricular ejection fraction; MadWT: mean absolute deviation of maximum segmental wall thickness; MaxWT: maximum end-diastolic wall thickness; MeanWT: mean end-diastolic wall thickness; LVMi: indexed left ventricular mass; MACE: major adverse cardiovascular event.

# Figure S4. Incident heart failure covariate residual *vs.* time plots.


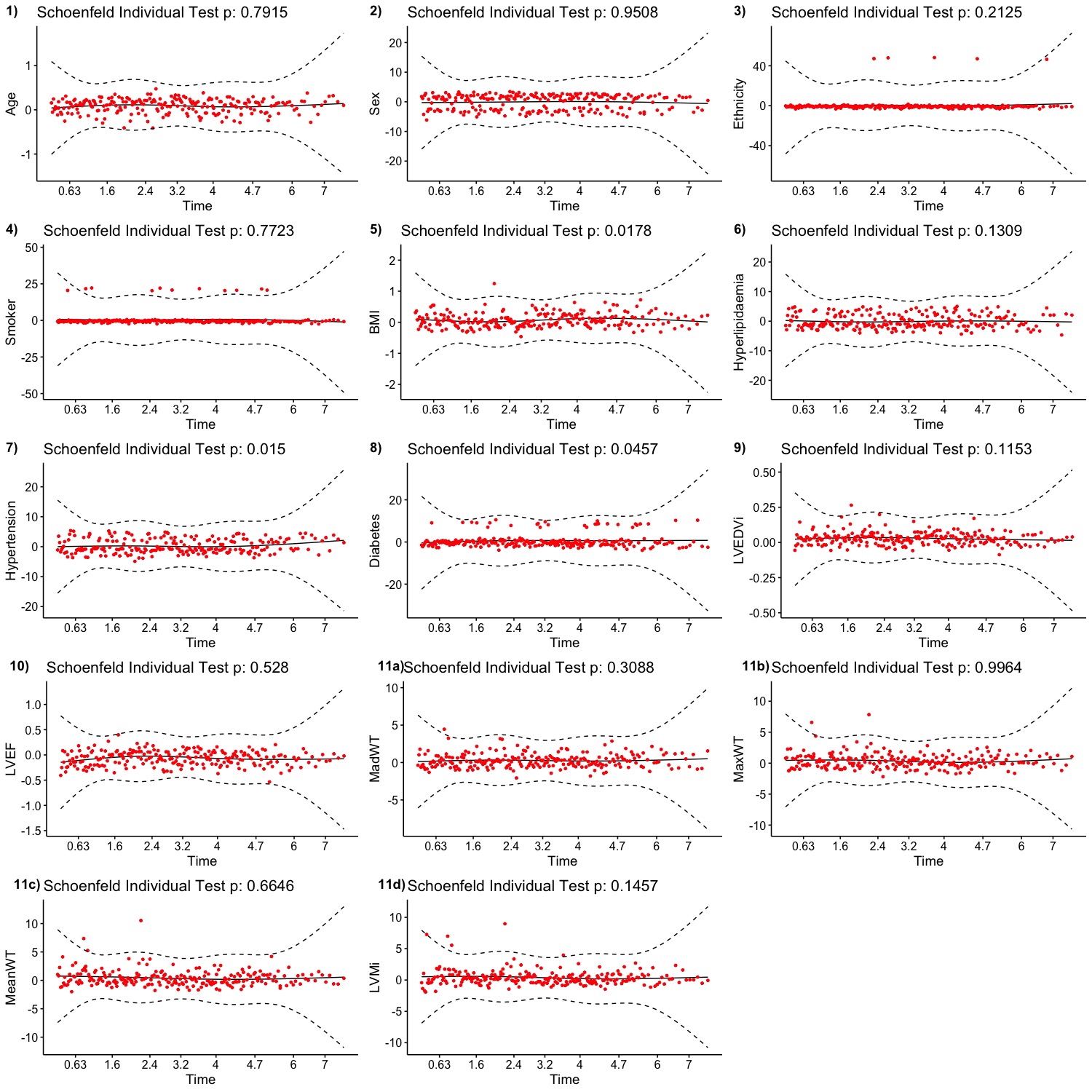


In the fully adjusted incident HF Cox regression model, assumption of proportional hazards is supported for *MadWT, MaxWT, MeanWT, LVMi* and their covariates graphically.

LVEDVi: indexed left ventricular end-diastolic volume; LVEF: left ventricular ejection fraction; MadWT: mean absolute deviation of maximum segmental wall thickness; MaxWT: maximum end-diastolic wall thickness; MeanWT: mean end-diastolic wall thickness; LVMi: indexed left ventricular mass; HF: heart failure. **Figure S5. Incident arrhythmia covariate residual *vs.* time plots.**


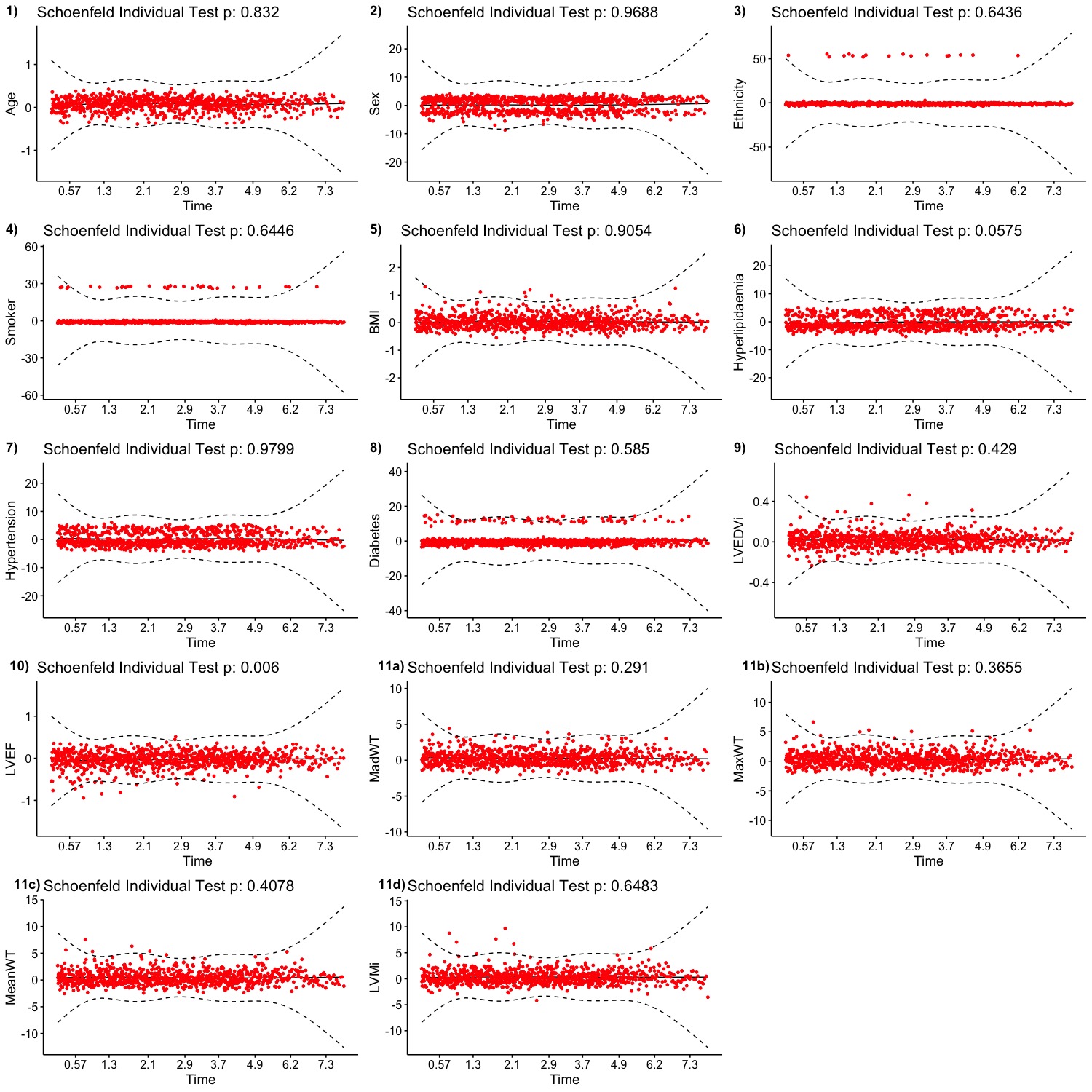


In the fully adjusted Cox regression model for incident arrhythmia events, assumption of proportional hazards is supported for *MadWT, MaxWT, MeanWT, LVMi* and their covariates graphically.

LVEDVi: indexed left ventricular end-diastolic volume; LVEF: left ventricular ejection fraction; MadWT: mean absolute deviation of maximum segmental wall thickness; MaxWT: maximum end-diastolic wall thickness; MeanWT: mean end-diastolic wall thickness; LVMi: indexed left ventricular mass.**Figure S6. All-cause death covariate residual *vs.* time plots.**


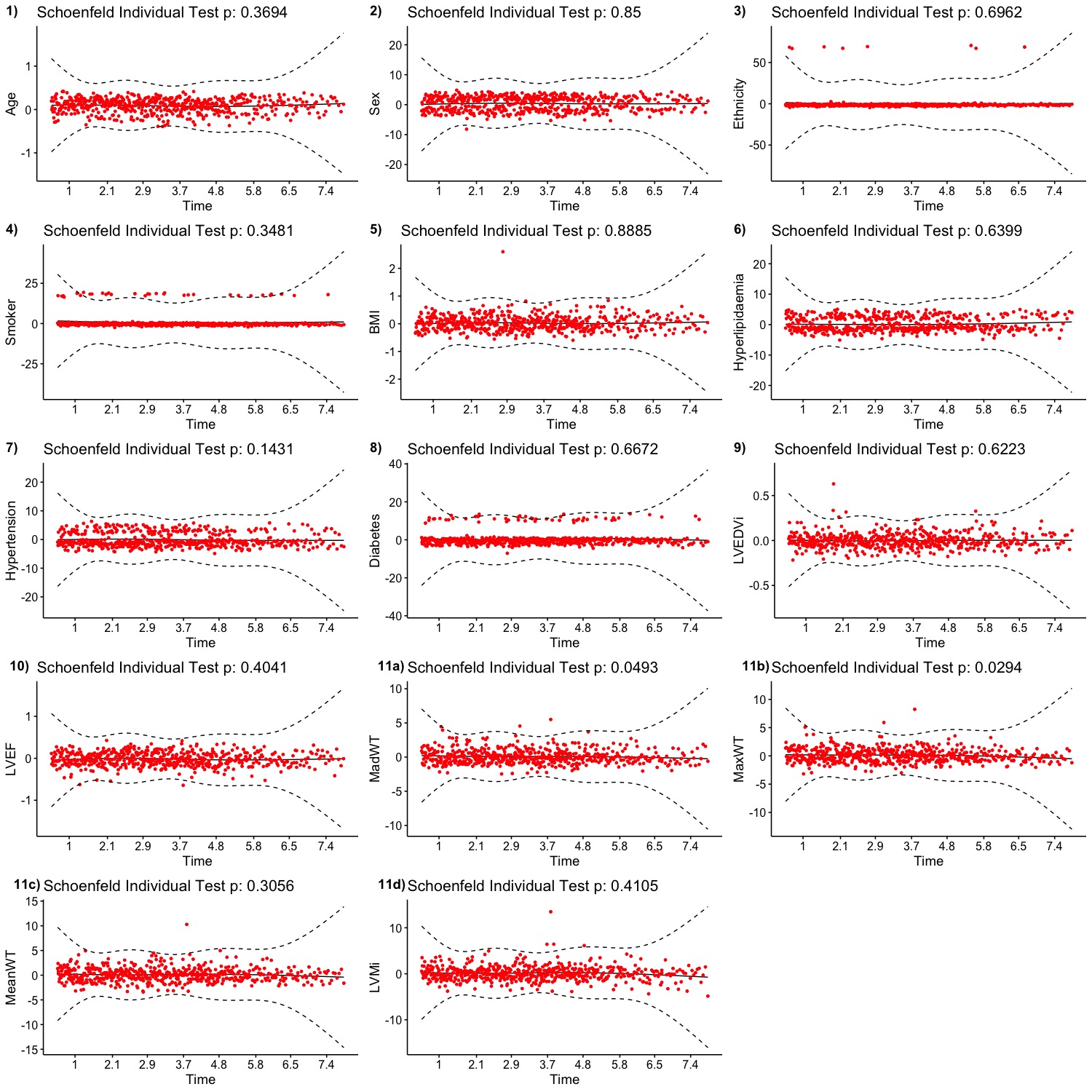


In the fully adjusted Cox regression model for death from any cause, assumption of proportional hazards is supported for *MadWT, MaxWT, MeanWT, LVMi* and their covariates graphically.

LVEDVi: indexed left ventricular end-diastolic volume; LVEF: left ventricular ejection fraction; MadWT: mean absolute deviation of maximum segmental wall thickness; MaxWT: maximum end-diastolic wall thickness; MeanWT: mean end-diastolic wall thickness; LVMi: indexed left ventricular mass.

# Figure S7. Incident myocardial infarction covariate residual *vs.* time plots.


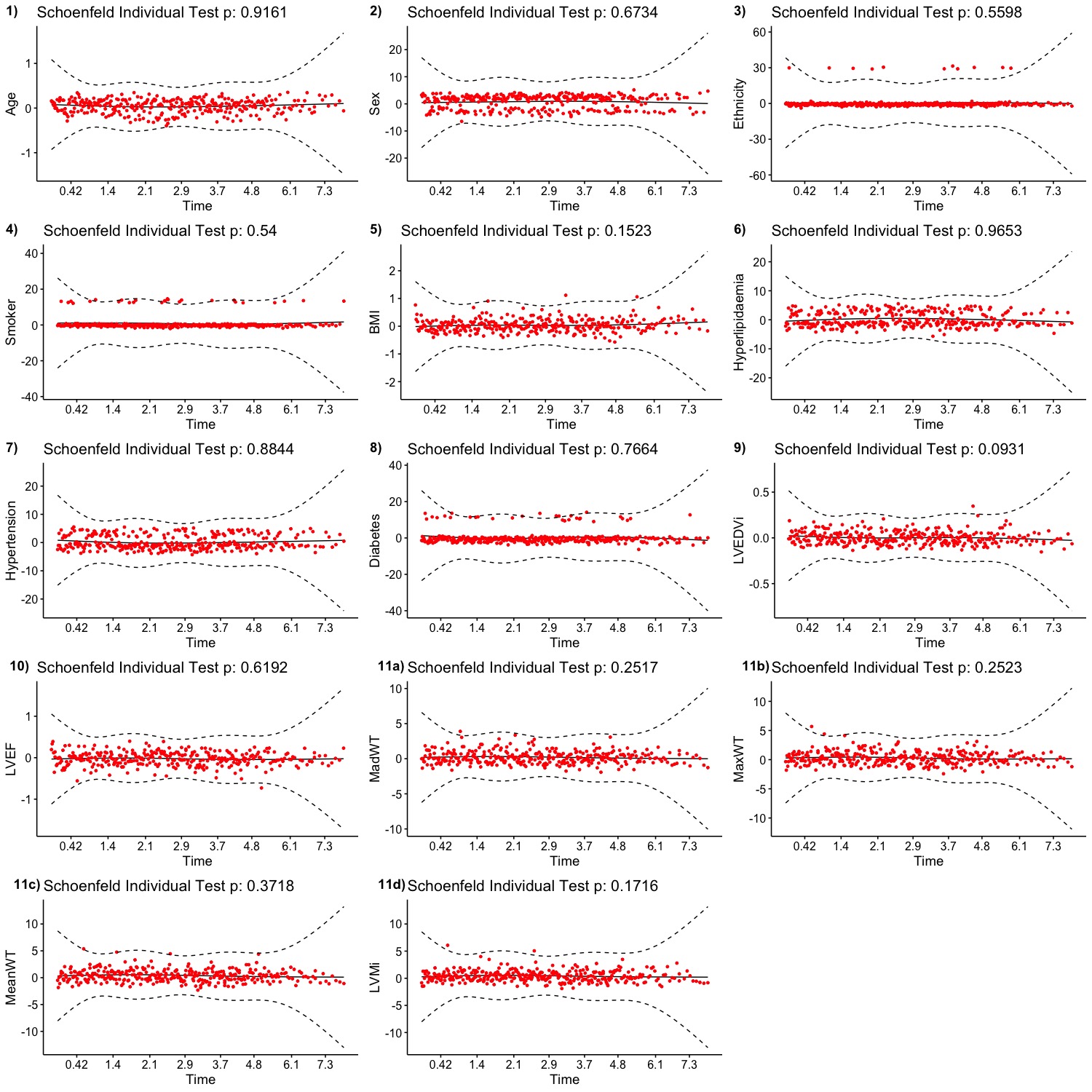


In the fully adjusted incident MI Cox regression model, assumption of proportional hazards is supported for *MadWT, MaxWT, MeanWT, LVMi* and their covariates graphically.

LVEDVi: indexed left ventricular end-diastolic volume; LVEF: left ventricular ejection fraction; MadWT: mean absolute deviation of maximum segmental wall thickness; MaxWT: maximum end-diastolic wall thickness; MeanWT: mean end-diastolic wall thickness; LVMi: indexed left ventricular mass; MI: myocardial infarction. **Figure S8. Incident stroke covariate residual *vs.* time plots.**


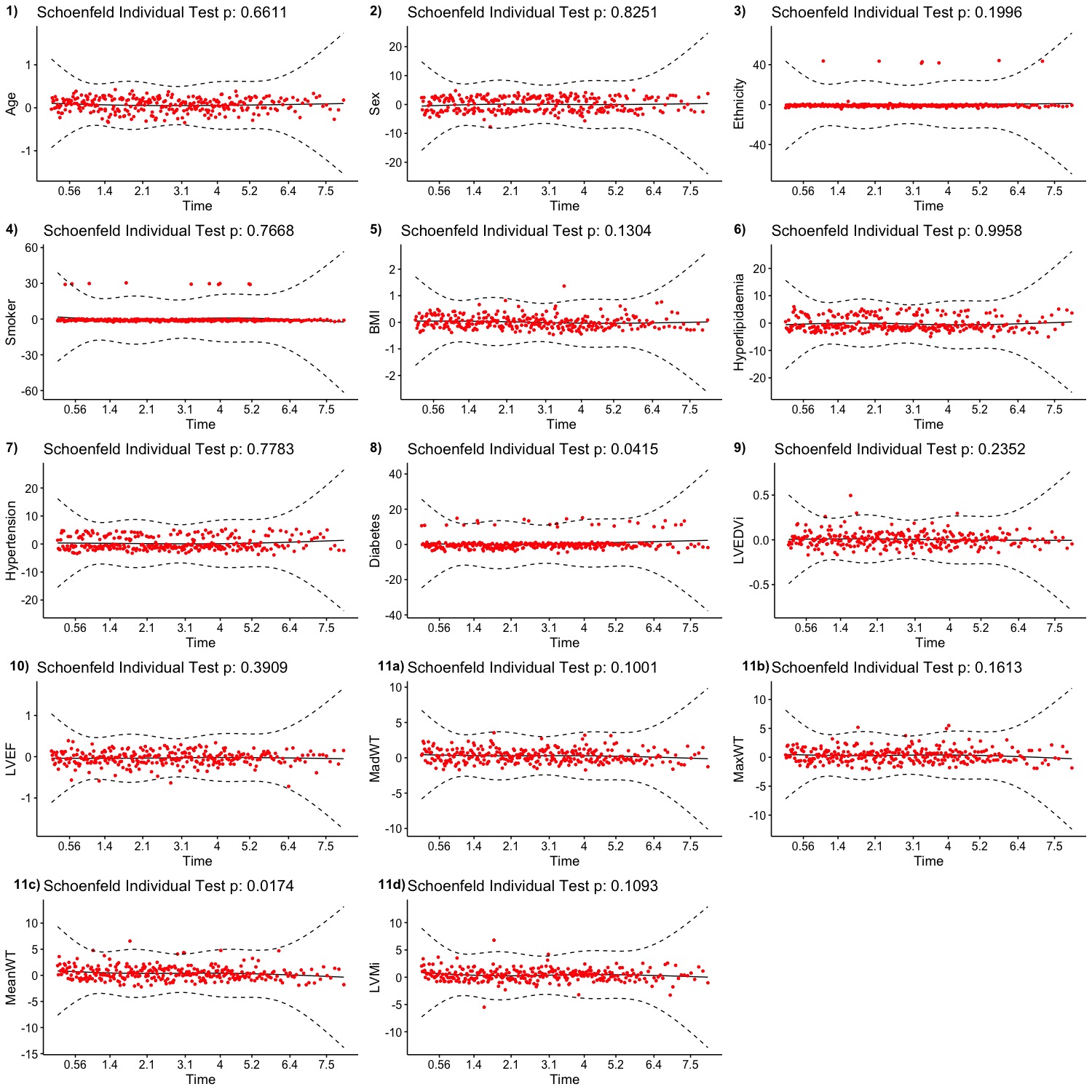


In the fully adjusted incident stroke Cox regression model, assumption of proportional hazards is supported for *MadWT, MaxWT, MeanWT, LVMi* and their covariates graphically.

LVEDVi: indexed left ventricular end-diastolic volume; LVEF: left ventricular ejection fraction; MadWT: mean absolute deviation of maximum segmental wall thickness; MaxWT: maximum end-diastolic wall thickness; MeanWT: mean end-diastolic wall thickness; LVMi: indexed left ventricular mass.

# Figure S9. CV death covariate residual *vs.* time plots.


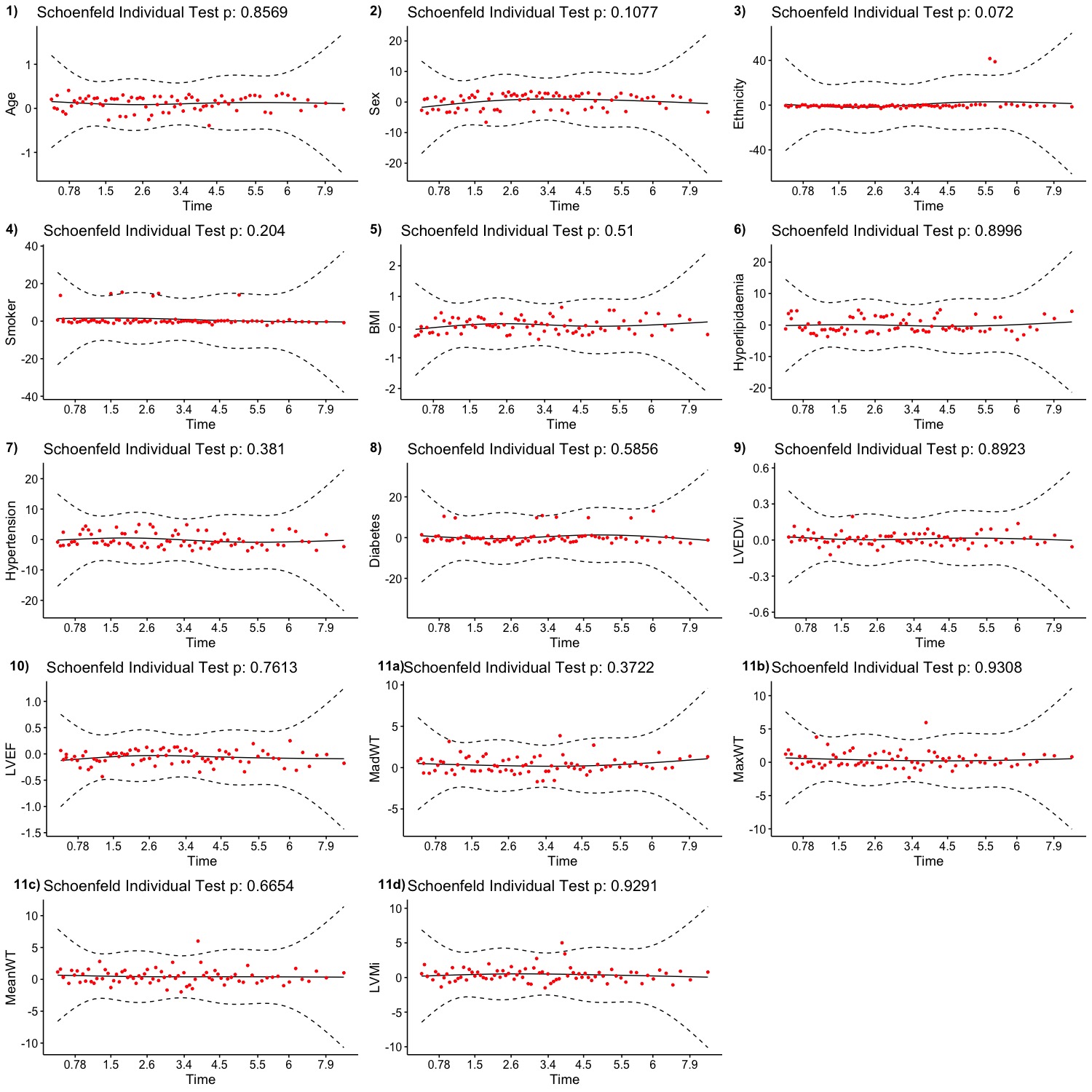


In the fully adjusted Cox regression model for CV death, assumption of proportional hazards is supported for *MadWT, MaxWT, MeanWT, LVMi* and their covariates graphically.

LVEDVi: indexed left ventricular end-diastolic volume; LVEF: left ventricular ejection fraction; MadWT: mean absolute deviation of maximum segmental wall thickness; MaxWT: maximum end-diastolic wall thickness; MeanWT: mean end-diastolic wall thickness; LVMi: indexed left ventricular mass; CV: cardiovascular.


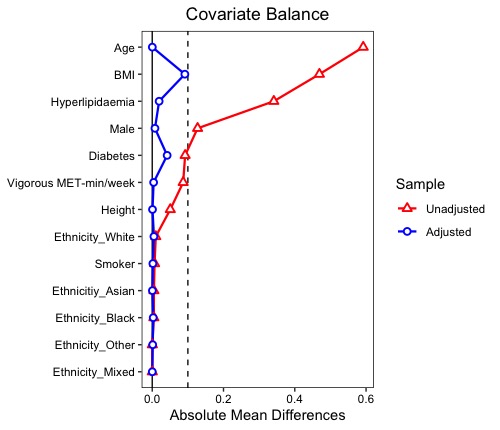

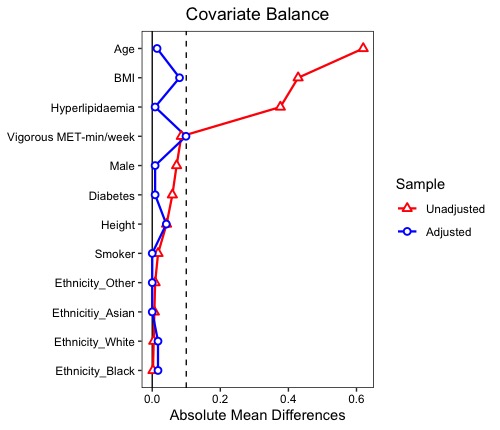

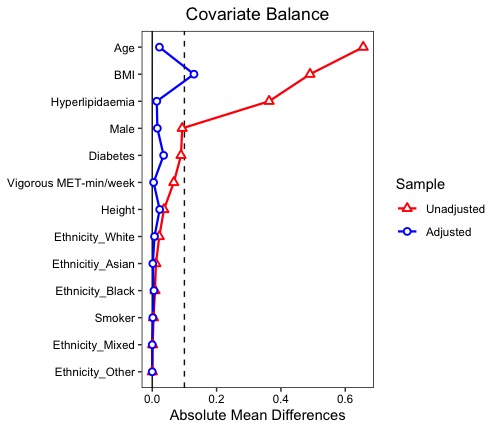

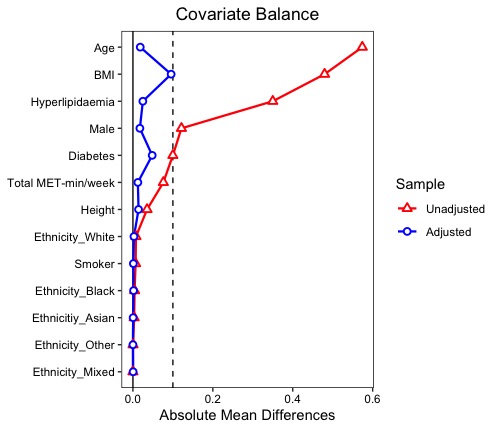

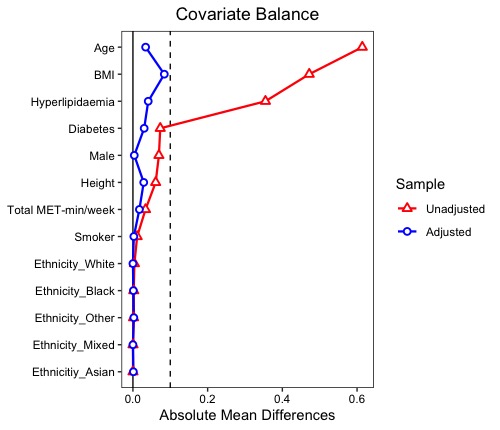

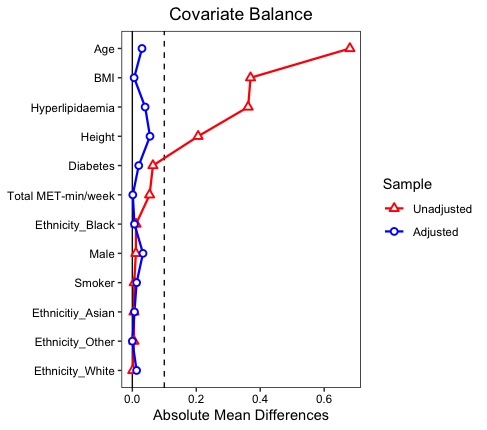

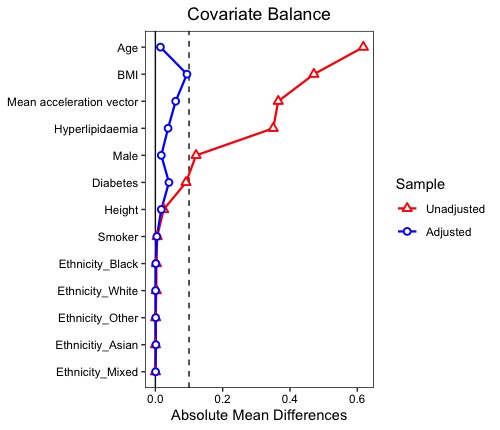

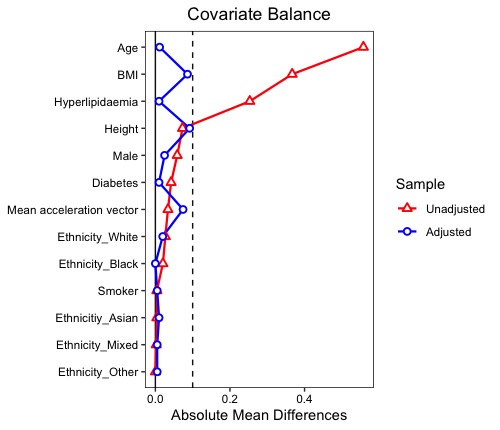

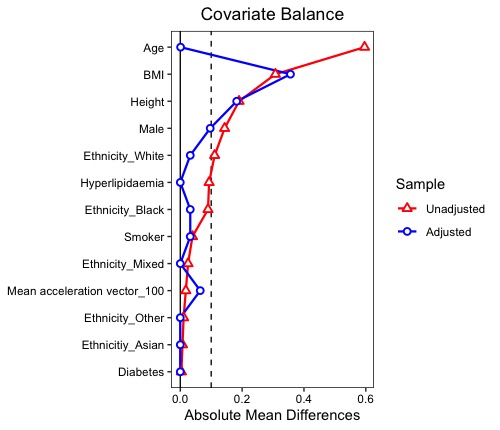


**A)** Vigorous MET-min/week

Unadjusted

Adjusted

**B)** Total MET-min/week

**C)** Accelerometry

**i)**

**ii)**

**iii)**

**i)**

**ii)**

**iii)**

**i)**

**ii)**

**iii)**

≥ 10%

5 ≤ %△ < 10

# Figure S10: Balance plots for PSM hypertensive and non-hypertensive cohorts.

In all-comers **(Panel i),** the top 10% **(Panel ii)** and top 1% **(Panel iii)** most physically active by vigorous MET-min/week (**Section A)**, total MET-min/week **(Section B)** and mean acceleration vector **(Section C),** cohorts were overall well-matched.

MET: metabolic equivalent of task; PSM: propensity score match.

# Figure S11. Study flowchart.

From 44,930 UKBB participants with CMR, 44,438 were included, dichotomized into healthy reference (n=19,531), CV risk factor (n=17,549) and disease (n = 7,358) cohorts.

UKBB: UK Biobank; CMR: Cardiovascular magnetic resonance imaging; IHD: ischemic heart disease; VHD: valvular heart disease; CKD: chronic kidney disease; CV: cardiovascular; BMI: body mass index.

# Figure S12. Forest plot, comparing WT indices to predict CV endpoints.

*MadWT, MaxWT*, *MeanWT* and *LVMi* were associated with a greater risk of MACE, heart failure and arrhythmias on Cox regression univariably and after adjusting for CV risk factors as well as established CMR biomarkers; they were only predictive of death from any cause on univariable analysis.

*Filled *vs.* unfilled marker indicates Bonferroni-corrected P<0.003 *vs.* ≥0.003.

MACE: major adverse cardiovascular event; MadWT: mean absolute deviation of maximum segmental wall thickness; MaxWT: maximum end-diastolic wall thickness; MeanWT: mean end-diastolic wall thickness; LVMi: indexed left ventricular mass; CVRF: cardiovascular risk factor; CMR: cardiovascular magnetic resonance imaging; SD: standard deviation; WT: wall thickness.

# Figure S13. Forest plot of WT indices & *LVMi* to predict MACE sub-endpoints.

*MadWT, MaxWT* and *MeanWT* and *LVMi* were associated with a greater risk of MI, stroke and CV death on Cox regression univariably and after adjusting for CV risk factors as well as established CMR biomarkers.

*Filled *vs.* unfilled marker indicates P < 0.05 *vs.* ≥ 0.05.

MACE: major adverse cardiovascular event; MadWT: mean absolute deviation of maximum segmental wall thickness; MaxWT: maximum end-diastolic wall thickness; MeanWT: mean end-diastolic wall thickness; LVMi: indexed left ventricular mass; CVRF: cardiovascular risk factor; CMR: cardiovascular magnetic resonance imaging; SD: standard deviation; WT: wall thickness.

# Figure S14. Forest plot of WT indices and *LVMi* to predict CV endpoints by sex.

Overall, *MadWT, MaxWT*, *MeanWT* and *LVMi* had larger effect sizes to predict MACE, HF, arrhythmia and death from any cause in men versus women.

*Filled *vs.* unfilled marker indicates P < 0.05 *vs.* ≥ 0.05.

MACE: major adverse cardiovascular event; MadWT: mean absolute deviation of maximum segmental wall thickness; MaxWT: maximum end-diastolic wall thickness; MeanWT: mean end-diastolic wall thickness; LVMi: indexed left ventricular mass; CVRF: cardiovascular risk factor; CMR: cardiovascular magnetic resonance imaging; SD: standard deviation; HF: heart failure; WT: wall thickness.

**B)**

**A)**

**C)**

# Figure S15. Relative difference of CMR-derived parameters by HTN status & PA level.

*MadWT* had the greatest relative difference between hypertension and no hypertension in PSM cohorts of all participants, the top 10% and 1% most physically active by total **(Panel A),** vigorous **(Panel B) MET**-min/week and mean acceleration vector **(Panel C)** respectively.

MadWT: mean absolute deviation of maximum segmental wall thickness; MaxWT: maximum end-diastolic wall thickness; MeanWT: mean end-diastolic wall thickness; LVMi: indexed left ventricular mass; LVEDVi: indexed left ventricular end-diastolic volume; LVMVR: left ventricular mass to volume ratio; LVEF: left ventricular ejection fraction; GLS: global longitudinal strain; HTN: hypertension; TPA: total physical activity; MET: metabolic equivalent of task; PA: physical activity; CMR: cardiovascular magnetic resonance imaging; WT: wall thickness.
